# Co-expression network analysis reveals transcription factors associated to cell wall biosynthesis in sugarcane

**DOI:** 10.1007/s11103-016-0434-2

**Published:** 2016-01-28

**Authors:** Savio Siqueira Ferreira, Carlos Takeshi Hotta, Viviane Guzzo de Carli Poelking, Debora Chaves Coelho Leite, Marcos Silveira Buckeridge, Marcelo Ehlers Loureiro, Marcio Henrique Pereira Barbosa, Monalisa Sampaio Carneiro, Glaucia Mendes Souza

**Affiliations:** Instituto de Química, Universidade de São Paulo, São Paulo, Brazil; Departamento de Biologia Vegetal, Universidade Federal de Viçosa, Viçosa, Brazil; Instituto de Biociências, Universidade de São Paulo, São Paulo, Brazil; Departamento de Fitotecnia, Universidade Federal de Viçosa, Viçosa, Brazil; Centro de Ciências Agrárias, Universidade Federal de São Carlos, São Carlos, Brazil; Universidade Federal do Recôncavo da Bahia, Cruz das Almas, Brazil

**Keywords:** Sugarcane, Expression profiling, Cell wall, Lignin, Co-expression network, Transcription factors

## Abstract

Sugarcane is a hybrid of *Saccharum officinarum* and *Saccharum spontaneum*, with minor contributions from other species in *Saccharum* and other genera. Understanding the molecular basis of cell wall metabolism in sugarcane may allow for rational changes in fiber quality and content when designing new energy crops. This work describes a comparative expression profiling of sugarcane ancestral genotypes: *S. officinarum*, *S. spontaneum* and *S. robustum* and a commercial hybrid: RB867515, linking gene expression to phenotypes to identify genes for sugarcane improvement. Oligoarray experiments of leaves, immature and intermediate internodes, detected 12,621 sense and 995 antisense transcripts. Amino acid metabolism was particularly evident among pathways showing natural antisense transcripts expression. For all tissues sampled, expression analysis revealed 831, 674 and 648 differentially expressed genes in *S. officinarum*, *S. robustum* and *S. spontaneum*, respectively, using RB867515 as reference. Expression of sugar transporters might explain sucrose differences among genotypes, but an unexpected differential expression of histones were also identified between high and low Brix° genotypes. Lignin biosynthetic genes and bioenergetics-related genes were up-regulated in the high lignin genotype, suggesting that these genes are important for *S. spontaneum* to allocate carbon to lignin, while *S. officinarum* allocates it to sucrose storage. Co-expression network analysis identified 18 transcription factors possibly related to cell wall biosynthesis while in silico analysis detected cis-elements involved in cell wall biosynthesis in their promoters. Our results provide information to elucidate regulatory networks underlying traits of interest that will allow the improvement of sugarcane for biofuel and chemicals production.

**Electronic supplementary material:**

The online version of this article (doi:10.1007/s11103-016-0434-2) contains supplementary material, which is available to authorized users.

## Introduction

Sugarcane is a C4 grass from the Saccharinae subtribe (Poacea family) that has the capacity to accumulate high levels of sucrose in its stems. Modern commercial sugarcane varieties are highly polyploidy and aneuploid (100–130 chromosomes arranged in 8–12 sets), resulted from the interspecific hybridization between *Saccharum officinarum* (2n = 80) and *Saccharumspontaneum* (2n = 36 − 128), with minor contributions from *S. robustum*, *S. sinense*, *S. barberi*, *Erianthus* and *Miscanthus* (Paterson et al. 2013). In general, the genomes of commercial varieties are mainly composed by chromosomes derived from *S. officinarum* (70–80 %), while a smaller portion of the composition is attributed to *S. spontaneum* (10–20 %) and to recombinant chromosomes from these two species (~10 %) (D’Hont 2005; D’Hont et al. 1996, 2008). These two main ancestral species show distinct phenotypes that were important in the breeding of the current varieties: *S. officinarum* is a sweet cane with thick, juicy and low-fiber culms, whereas *S. spontaneum* typically exhibits a low sugar content, thin and fibrous culms, more tillers per plant, and higher stress tolerance (Paterson et al. 2013).

For decades, the sugarcane industry has been using sugar-rich juice from stalks to produce ethanol via fermentation and employing the residual biomass (bagasse) to produce electricity through burning, a process referred to as co-generation, placing sugarcane among the best alternatives for bioenergy production (Souza et al. 2014). Moreover, new technologies are becoming available to produce bioethanol from bagasse, also known as cellulosic bioethanol, during which the carbohydrates from the bagasse cell wall are hydrolyzed, and simple sugars are released for fermentation (Amorim et al. 2011). The possibility of using biomass for bioenergy production, and the recent interest in bioenergy-dedicated crops, has led to increasing interest in the production of a cane that generates the maximum amount of primary energy per hectare, referred to as energy cane, which typically exhibits a lower sugar content, but higher biomass yield and higher fiber content (Leal et al. 2013).

In order to improve the production of bioenergy from cane-derived sources, it is important to understand better the biosynthesis of the sugarcane cell wall, as it may allow for plants with increased biomass and cell walls that are more amenable to hydrolysis. Nevertheless, several factors must be taken into account to produce varieties with these characteristics. Special attention must be given to cell wall recalcitrance. Plant cell walls evolved to avoid pathogen attack, to ensure plant stiffness, and to reduce water loss. Lignin, one of the main components of the cell wall, is a heterogeneous hydrophobic polymer that is covalently crosslinked to hemicellulose, conferring strength and rigidity (Boerjan et al. 2003; Carpita and Gibeaut 1993). These characteristics, while important for plant growth and productivity, hamper bagasse hydrolysis and cellulosic ethanol production. Therefore, lignin is thought to be one of the causes of cell wall recalcitrance (Himmel et al. 2007). In addition to lignin, other phenolic compounds are also thought to be important for recalcitrance, such as ferulic acid and coumaric acid, which are characteristic of the cell wall of grasses and can be important for crosslinking lignin to hemicellulose (de O. Buanafina 2009; Harris and Trethewey 2009; Molinari et al. 2013; Ralph et al. 1995; Vogel 2008).

Fiber content is another important trait for increasing biomass yield (Leal et al. 2013). However, sugarcane breeding has been mainly focused on sugar content, and fiber has been weighted negatively in some selection indices (Wei et al. 2008), which may explain why current elite cultivars and germplasms accumulate high sucrose, but do not perform well in total biomass accumulation. Moreover, commercial sugarcane varieties exhibit a relatively narrow genetic base (Lima et al. 2002; Ming et al. 2006; Roach 1989), which might lead to the reduction of the yield gains achieved in each new commercial variety (Dal-Bianco et al. 2012), even though the theoretical and experimental maximums for cane yield are far larger than the current average yield (Waclawovsky et al. 2010).

Under this scenario, the introgression of ancestral genotypes into breeding programs to broaden the genetic background and increase fiber and biomass contents is already becoming a reality, even though there is limited knowledge about the molecular mechanisms that regulate these traits. Most of the molecular studies on ancestral sugarcane species focus on the identification of molecular markers and polymorphisms (Aitken et al. 2007; Berkman et al. 2014; Bundock et al. 2012; Li et al. 2011; Ming et al. 2002; Silva et al. 1993; Zhang et al. 2013), genotyping and phylogeny (Chang et al. 2012; Khan et al. 2009; Pan et al. 2000; Takahashi et al. 2005), chromosome mapping (D’Hont et al. 1996; Ha et al. 1999; Piperidis et al. 2010), miRNAs (Zanca et al. 2010), and transposon-related sequences (Rossi et al. 2004). Moreover, although transcriptomic studies on hybrid sugarcane cultivars are abundant [reviewed by (de Siqueira Ferreira et al. 2013; Manners and Casu 2011)], studies focusing on cell wall metabolism are far more scarce: Lima and colleagues estimated the composition of the cell wall on the basis of expression patterns (Lima et al. 2001), whereas Casu and colleagues identified clusters of cell wall-related genes that had differential expression along the internodes (Casu et al. 2007).

In this study, we carried out a transcriptome analysis of three ancestral genotypes and one commercial variety, and constructed a co-expression network to identify target genes (especially transcription factors), regulatory networks, and promoters that might be useful for sugarcane and energy cane improvement. We focused our analysis on the identification of cell wall-related genes in an attempt to advance the understanding of the particularities of cell wall biosynthesis and regulation in sugarcane, producing knowledge to contribute to the development of strategies to increase cane biomass yields and to design an energy cane that is better suited for industrial needs. This report describes a large-scale transcriptomic analysis of sugarcane ancestral genotypes that allowed the identification of possible gene networks involved in cell wall metabolism.

## Methods

### Plant material

Plants of the *S. officinarum* (caiana listrada), *S. robustum* (IM76-229) and *S. spontaneum* (IN84-058) genotypes and the commercial hybrid RB867515 were grown in a field in single rows of 5 m using standard sugarcane cultivation practices from October 2010 to July 2011, when samples and physiological data were collected (9-month-old plants). Three biological replicates for each genotype were harvested (two replicates for microarray analysis and one replicate for qPCR validation). Leaves and immature, intermediate and mature internodes were cut and immediately frozen in liquid nitrogen, then kept in dry ice until the being properly stored in ultra-low temperature freezers. The immature internodes consisted of a pool of the two uppermost internodes (internodes 1 and 2), near the apical meristem, while the intermediate and mature internodes were the fifth and the ninth internodes, respectively, counting from the immature internodes, as described in (Papini-Terzi et al. 2009). The leaf samples consisted only of the uppermost visible collar (dewlap) leaf (leaf+1). Plants were cut, and ratoon plants were sampled (three biological replicates) for qPCR analysis (Online Resource 4) when the plants were 7 months old (grown from August 2011 to March 2012).

### Morpho-physiological data

Photosynthetic and transpiration data were collected using an InfraRed Gas Analyzer (IRGA) (LCi Portable Photosynthesis System; ADC Bioscientific, Hoddesdon, UK) in the field from 11 a.m. to 1 p.m. under ambient temperature, CO_2_ and water vapor conditions before the plants were harvested. Solar light (ambient) was used as the light source, and the photon flux density ranged from 1100 to 1300 μmol m-2 s-1. Measurements were carried out in four biological replicates and two technical replicates from the middle portion of the leaf+1. Brix content, plant height and culm mass were measured in three biological replicates. The Brix content of the sugarcane stalk was measured with a portable refractometer (N1 model, ATAGO, Japan).

### Histochemical analysis

Samples were harvested and fixed in a solution of FAA50 (formaldehyde, glacial acetic acid and ethanol 50 %; 5:5:90, v/v) under vacuum for 48 h. Hand-cut section (40–60 µm) were stained with phloroglucinol as described in (Patten et al. 2005). Photographs were taken using an Olympus BX51 light microscope and Olympus Evolt E-330 camera within 10 min of staining. The presented figures show a representative image of three biological replicates.

### Lignin analysis

Lignin analysis was carried out by the Complex Carbohydrate Research Center at the University of Georgia. A lyophilized sample was weighed (4–5 mg) in a small sample cup, then pyrolyzed using a Pyrolyzer at 500 °C, and the residues were analyzed using a Molecular Beam Mass Spectrometer, where all lignin residues were identified from their mass profile. Each sample was analyzed in duplicate runs. The uncorrected lignin content was calculated through multivariate analysis (principal component analysis) and corrected using NIST Sugarcane Bagasse (Lignin = 24.4 % of dry weight). The results were subjected to one-way ANOVA and Tukey’s posttest (*p* < 0.05).

### Glucose, fructose and sucrose analyses

Approximately 10 mg of a lyophilized sample was weighed in a 2 mL microcentrifuge tube, and soluble sugars were extracted by incubating the samples with 1.5 mL of 80 % ethanol at 80 °C for 20 min. After incubation, the samples were centrifuged for 10 min at 12,000×*g*, and the supernatant was collected. This procedure was repeated for a total of five washes, collecting the supernatant each time. The supernatant was dried under vacuum (Speed-vac^®^) and resuspended in 1 mL of deionized water. Liposoluble pigments were extracted from leaf samples with 0.5 mL of chloroform, followed by incubation for 5 min at room temperature and centrifugation for 5 min at 12,000×*g*. The aqueous phase was collected for analysis. Aliquots of each sample were analyzed through high-performance anion-exchange chromatography (HPAEC/PAD), using a Carbopac PA1 column eluted with sodium hydroxide 100 mM at a constant flow rate of 1 mL/min in the Dionex-ICS3000^®^ system (de Souza et al. 2013). Sucrose, fructose and glucose standards were used at concentrations 50, 100 and 200 mM to calculate the equivalent quantities in the samples.

### RNA extraction

RNA was isolated using the TRIzol reagent (Invitrogen) according to the manufacturer’s instructions, followed by treatment with DNase I (Invitrogen) and cleanup using the RNeasy mini kit (Qiagen). RNA integrity and concentration were evaluated in a NanoDrop 1000 spectrophotometer (Thermo Scientific) and via Agilent 2100 Bioanalyzer electrophoresis with the Agilent RNA 6000 Pico Kit (Agilent Technologies).

### Oligoarray hybridization and data analysis

Two biological replicates and dye swaps were used for each experiment. The procedures for oligoarray design, cRNA labeling, hybridization and data processing, normalization and analysis are described in detail in (Lembke et al. 2012). Briefly, labeling and hybridization were conducted following the Two-color Microarray-Based Gene Expression Analysis protocol (Low input Quick Amp Labeling, Agilent Technologies), and the slides were scanned using a GenePix 4000B scanner (Molecular Devices, Sunnyvale, CA, USA). Data were extracted using Feature Extraction 9.5.3.1 software (Agilent Technologies), and normalization was performed in two steps, using non-linear LOWESS normalization (Yang et al. 2002) and a modified HTself method (Vencio and Koide 2005) adapted for the Agilent platform. A gene was only considered up-/down-regulated if 96 % confidence (*p* value <0.04) was achieved for each reference set based on the modified HTself method, and a gene was considered differentially expressed only when at least 70 % of the spots for this gene model showed the same expression profile in a given experiment, as defined under the HTself method. For hierarchical clustering of all significantly expressed genes, log2 expression values were normalized through median centering, after which we used Spearman’s correlation for samples and Pearson’s correlation for genes to construct hierarchical clusters. For cell wall-related genes, we performed hierarchical clustering for samples and genes using log2 expression values and Pearson’s correlation. Functional category enrichment was assessed using the GeneMerge tool (Castillo-Davis and Hartl 2003) as described in (Lembke et al. 2012).

### Quantitative real-time PCR (qPCR)

cDNA was synthesized using SuperScript III First-Strand Synthesis SuperMix (Invitrogen) with oligo(dT) primers according to the manufacturer’s instructions. Gene-specific primers were designed with Primer Express software (Applied Biosystems), and qPCR assays were carried out using Fast SYBR Green Master Mix (Applied Biosystems) and the 7500 Fast Real-Time PCR System (Applied Biosystems) under standard protocols. The results were analyzed with qBase 2.0 software (Biogazelle, Zwijnaarde, Belgium) as described in (Hellemans et al. 2007) using the geometric mean of at least the two most stable endogenous controls (reference targets) selected by geNorm software (within qBase) among a set of five endogenous controls tested (polyubiquitin, GAPDH, 60S ribosomal protein subunit, actin and tubulin) as a normalization factor. The results were subjected to one-way ANOVA and Tukey’s posttest (*p* < 0.05).

### KEGG pathway activity score

Pathway activity (PA) score analysis was carried out as described in (Nishiyama et al. 2014). Briefly, SUCEST ESTs were mapped against the KEGG database, and each SAS received a KEGG Orthology (KO) identifier, which assigned each SAS to its respective pathway. Thus, the expression values of each SAS were allocated to the respective pathways. All expression values for a given pathway formed by different SAS were summed and normalized based on the proportion of detected antisense transcripts for the respective pathway. Then, the scores were log2-transformed and normalized via median centering. Hierarchical clustering was constructed using the average method and Spearman correlation.

### Datamining for cell wall-related genes

We searched for cell wall-related genes in the SUCEST database using two cell wall gene catalogues as a reference: Cell Wall Genomics (Yong et al. 2005) and Maizewall (Guillaumie et al. 2007). The obtained sugarcane genes were then manually re-annotated to produce a sugarcane cell wall catalogue with 1606 sequences, of which 541 are present in the sugarcane Agilent array.

### Co-expression analysis

We used only sense expression data from immature and intermediate internodes, employing each biological and technical replicate as an individual dataset (totaling 24 samples) to construct a co-expression network with the WGCNA R-package (Langfelder and Horvath 2008) and the following parameters: power = 8; merge Cut Height = 0.15, weight threshold = 0.25. Then, we searched for each module for genes of interest (*4cl* from lignin biosynthesis) and filtered them by cell wall-related genes and transcription factors. The network was then visualized in Cytoscape software (Saito et al. 2012). TFs nomenclature was based on the Grassius database (Yilmaz et al. 2009).

### In silico promoter analysis

We used BLASTn to search for candidate promoter sequences for each chosen sugarcane EST (SAS) against a sugarcane BAC genome database (De Setta et al. 2014) and then selected 2 kb upstream of the 5′-UTR. Subsequently, candidate promoter sequences were subjected to a search for cis-elements related to cell wall biosynthesis, i.e., SNBEs (Zhong et al. 2010) and SMREs (Zhong and Ye 2012), using the TOMTOM (Gupta et al. 2007) and FIMO (Grant et al. 2011) tools and employing the consensus sequences of each cis-element as inputs for searches, using a *p* value threshold of 0.001.

## Results and discussion

### *Saccharum spontaneum* shows markedly different phenotype

Physiological and morphological parameters of the commercial sugarcane variety RB867515 and the ancestral sugarcane genotypes *S. officinarum*, *S. robustum* and *S. spontaneum* were measured (Table 1) in order to identify contrasting groups. Two groups could be separated based on sugar content (Brix° and soluble sugar analysis in mature internodes, Table 1): high Brix° (RB867515 and *S. officinarum*) and low Brix° (*S. robustum* and *S. spontaneum*). RB867515 had 3–7 times more fresh biomass (1.56 kg) than the ancestral genotypes (Table 1). In addition, *S. spontaneum* clearly differed from the other genotypes, as it had lower culm diameter, water content in the culm, photosynthetic rate, transpiration, sugar contents in intermediate and mature internodes, and had increased water use efficiency and lignin content in intermediate and mature internodes (Table 1). Moreover, despite having the lowest fresh weight in the culm, *S. spontaneum* produced a high dry biomass yield, mainly because plants of this species had 20 % less water content in the culm than RB867515 (Table 1) and were able to produce a greater number of tillers, resulting in more dry biomass per unit area. *S. robustum* is at an intermediate level between *S. spontaneum* and *S. officinarum*, in several of its characteristics, including culm diameter, water content, and sugar content in mature internodes (Table 1).

**Table 1:** Physiological and morphological measurements

Characteristic	Genotype			
	RB867515	*S. officinarum*	*S. robustum*	*S. spontaneum*
Culm diameter (mm)	20.15 ± 0.15^a^	25.94 ± 1.34^b^	15.22 ± 0.10^c^	8.75 ± 0.16^d^
Water content in the culm (%)	83.7 ± 0.8^ab^	87.0 ± 0.8^a^	78.8 ± 0.8^b^	63.1 ± 2.5^c^
Culm fresh weight (kg)	1.56 ± 0.045^a^	0.43 ± 0.009^b^	0.28 ± 0.013^c^	0.22 ± 0.009^c^
Plant height (m)	2.35 ± 0.028^a^	0.87 ± 0.029^b^	1.25 ± 0.057^c^	2.42 ± 0.037^a^
Brix°	10.67 ± 0.33^a^	10.67 ± 0.33^a^	5.67 ± 0.33^b^	2.26 ± 0.26^c^
Photosynthesis (A) (µmol CO_2_ m^−2^ s^−1^)	23.5 ± 1.2^a^	27.5 ± 2.0^a^	23.4 ± 1.4^a^	15.1 ± 1.1^b^
Transpiration (E) (mmol H_2_O m^−2^ s^−1^)	6.7 ± 0.22^a^	7.0 ± 0.23^a^	6.6 ± 0.55^a^	2.9 ± 0.44^b^
Water use efficiency (WUE) (A/E)	3.5 ± 0.11^a^	3.8 ± 0.25^a^	3.6 ± 0.29^a^	5.3 ± 0.47^b^
Stomatal conductance	0.21 ± 0.013^a^	0.24 ± 0.016^a^	0.18 ± 0.030^ab^	0.10 ± 0.016^b^
Lignin content (% of dry weight)				
Leaf+1	17.4 ± 0.06^a^	17.8 ± 0.11^a^	16.8 ± 0.52^a^	17.4 ± 0.36^a^
Immature internodes	13.8 ± 0.36^ab^	14.5 ± 0.30^b^	13.3 ± 0.26^ab^	12.9 ± 0.39^a^
Intermediate internodes	12.2 ± 0.37^a^	12.9 ± 0.58^a^	13.2 ± 0.44^a^	16.8 ± 0.21^b^
Mature internodes	16.6 ± 0.15^a^	14.8 ± 0.43^a^	16.5 ± 0.73^a^	19.3 ± 0.78^b^
Soluble sugars (mg/g of dry weight)				
Sucrose				
Leaf+1	33.4 ± 1.13^a^	31.5 ± 0.18^a^	31.3 ± 1.32^a^	35.9 ± 2.29^a^
Intermediate internodes	34.5 ± 4.11^a^	46.8 ± 2.41^a^	42.3 ± 3.51^a^	44.3 ± 3.02^a^
Mature internodes	78.3 ± 0.85^a^	61.3 ± 1.98^b^	57.9 ± 1.29^b^	39.8 ± 1.92^c^
Reducing sugars (glucose + fructose)				
Leaf+1	5.9 ± 1.55^a^	15.8 ± 0.84^b^	15.2 ± 1.83^bc^	9.3 ± 0.95^ac^
Intermediate internodes	147.9 ± 5.85^a^	136.8 ± 17.38^a^	98.8 ± 16.75^ab^	58.7 ± 4.66^b^
Mature internodes	158.9 ± 9.5^a^	164.1 ± 6.42^a^	81.3 ± 9.43^b^	28.7 ± 3.82^c^
Total (sucrose + glucose + fructose)				
Leaf+1	39.1 ± 2.68^a^	47.3 ± 0.65^a^	46.5 ± 2.04^a^	45.2 ± 3.11^a^
Intermediate internodes	182.2 ± 9.76^a^	183.6 ± 16.89^a^	141.1 ± 20.13^ab^	103.1 ± 7.66^b^
Mature internodes	237.2 ± 9.27^a^	225.4 ± 6.04^a^	139.2 ± 8.47^b^	68.1 ± 4.25^c^

Different letters next to of each value denote different means between genotypes by one-way ANOVA followed by Tukey’s test (*p* < 0.05). Differences between tissues were not evaluated. Error = SEM; N = 3 for all measurements, except for photosynthesis, transpiration and WUE, where N = 8

**Fig. 1:**
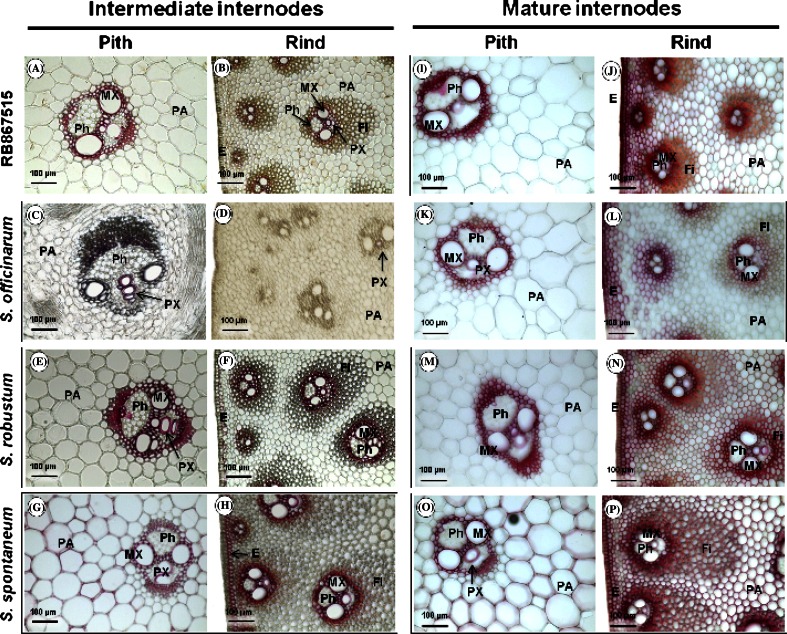
Phloroglucinol staining for lignin detection (*red*) in intermediate and mature internode cross-sections in the pith and rind regions. RB867515: **a**, **b**, **i** and **j**; *S. officinarum*: **c**, **d**, **k** and **l**; *S. robustum*: **e**, **f**, **m** and **n**; *S. Spontaneum*: **g**, **h**, **o**, and **p**. *MX* metaxylem, *PX* protoxylem, *Ph* phloem, *Fi* fibers, *E* epidermis, *PA* parenchyma

Lignin deposition in the intermediate and mature internodes was found to differ among genotypes (Fig. 1). In *S. officinarum*, lignin deposition was mainly restricted to the tracheary elements (meta and protoxylem) (Fig. 1c, d), whereas the lignin was deposited in the xylem and surrounding of the entire vascular bundle in the other genotypes (Fig. 1a, b, e–h). Additionally, the staining was stronger in the pith parenchyma and sub-epidermal parenchymatic cells of *S. spontaneum* (Fig. 1g, h), and lignified fibers associated with the vascular bundle in this genotype were also longer (Fig. 1p), which correlates with the higher lignin content of this genotype.

### Microarray data shows that RB867515 and *S. officinarum* have similar expression profiles

We carried out microarray experiments in three tissues: the leaf+1 and immature and intermediate internodes in order to identify genes that could be responsible for the physiological and morphological differences among genotypes. A customized sugarcane oligoarray (Agilent Technologies, Santa Clara, CA, USA), which comprises of 21,902 probes for the detection of 14,522 sugarcane assembled sequences (SAS) in sense orientation and 7380 probes for antisense transcript detection, was used for all hybridizations (Lembke et al. 2012). We identified 13,616 probes that showed expression signal above background in at least one tissue/genotype, which represents 62.2 % of the probes on the array. Most of these were sense probes, as 86.9 % of all sense probes (12,621 out of 14,522) but only 13.5 % for of the antisense probes (995 out of 7380) showed expression signal above background (Table 2). Interestingly, we also observed SAS that had only antisense expression (110–197 probes). Assuming that 33,000 is the approximate total number of genes in sugarcane (Yilmaz et al. 2009), 38 and 3 % of all sugarcane genes were detected expressing sense and antisense transcripts, respectively.

**Table 2:** Number of probes showing an expression signal above background in each genotype and tissue

	Probes with expression signal	Sense probes	Antisense probes	SAS with only sense expression	SAS with only antisense expression	SAS with sense and antisense expression
Leaf+1						
RB867515	11,418	10,913	505	10,583	175	330
*S. officinarum*	10,616	10,215	401	9976	162	239
*S. robustum*	10,278	9947	331	9733	117	214
*S. spontaneum*	10,318	9970	348	9732	110	238
Immature internodes						
RB867515	12,023	11,488	535	11,150	197	338
*S. officinarum*	11,687	11,162	525	10,827	190	335
*S. robustum*	10,820	10,472	348	10,271	147	201
*S. spontaneum*	11,274	10,829	445	10,535	151	294
Intermediate internodes						
RB867515	11,402	10,939	463	10,651	175	288
*S. officinarum*	11,135	10,745	390	10,522	167	223
*S. robustum*	10,681	10,317	364	10,103	150	214
*S. spontaneum*	9976	9660	316	9479	135	181
Total Unique Probes*	13,616 (62.2 %)^a^	12,621 (86.9 %)^b^	995 (13.5 %)^c^	–	–	–

* Percentages in relation to each type of probe spotted on slide

^a^Total (21,902 probes)

^b^Sense (14,522 probes)

^c^Antisense (7380 probes)

**Fig. 2:**
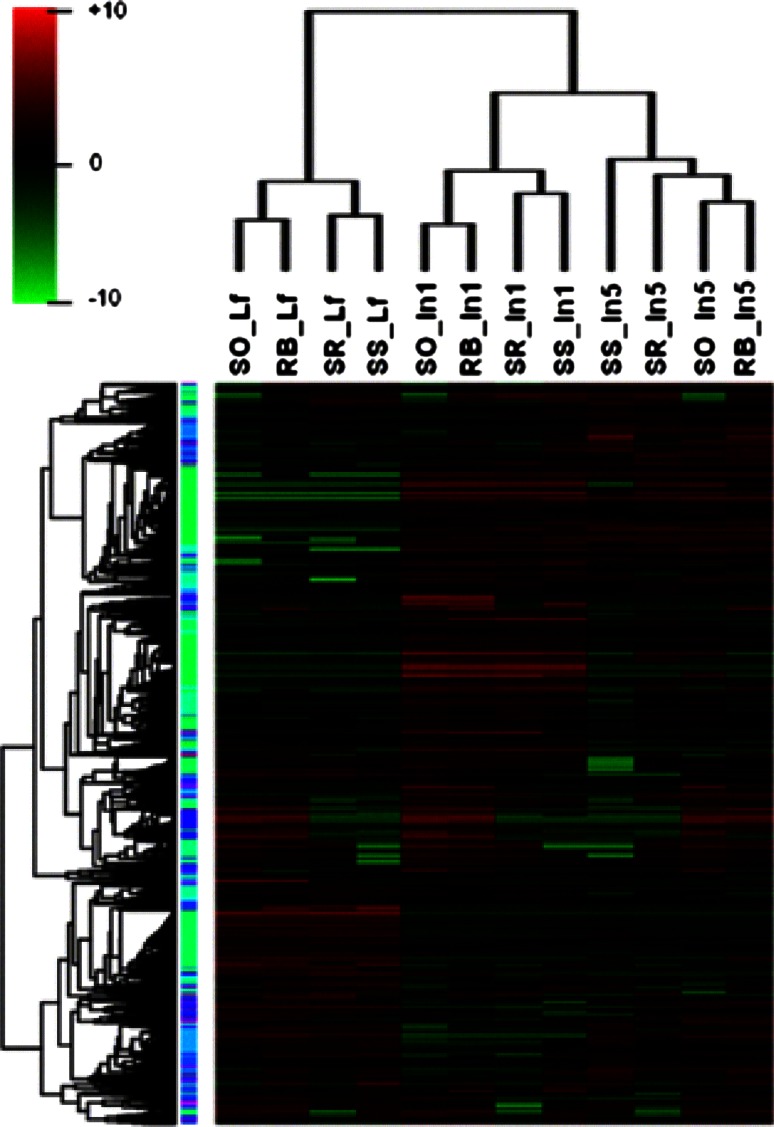
Hierarchical clustering of all expressed genes in the sugarcane ancestral and hybrid genotypes using normalized log2 expression data. The samples are indicated as follows (genotype_tissue): RB, RB867515; SO, *S. officinarum*; SR, *S. robustum*; SS, *S. spontaneum*; In1, immature internodes; In5, intermediate internodes; Lf, leaf

Heat mapping and hierarchical clustering analysis showed that the organs from different genotypes could be separated by their expression profiles (Fig. 2). Moreover, the hybrid RB867515 and *S. officinarum* were grouped together for all three analyzed tissues, which could be a reflection of the 70–80 % of the sugarcane hybrid genome that is shared between the commercial sugarcane varieties and *S. officinarum* (D’Hont 2005; D’Hont et al. 1996, 2008). This type of correlation between expression diversity and genetic variation has also been observed in sorghum (Jiang et al. 2013). Different sorghum lines had their transcriptome analyzed and correlated to genomic variations (SNPs, Indels, structural variations) leading the authors to conclude that the difference in gene expression is determined by the divergence at the genomic level (Jiang et al. 2013; Shakoor et al. 2014). We further searched for enrichment of functional categories in each genotype in the expressed genes using the GeneMerge tool (Castillo-Davis and Hartl 2003) (Online Resource 1). Among the most significantly enriched categories (e-score = 0), “Oxidative Phosphorylation”, “Light Harvesting”, and “Cytoskeleton and Vesicle Trafficking” were present in all genotypes. The high Brix° plants, *S. officinarum* and RB867515, showed similar profiles, sharing the categories “RNA Metabolism”, “Protein Metabolism” and “Circadian Clock” with e-score = 0, suggesting that they also have similar expression profiles, as mentioned above. However, only RB867515 exhibited “DNA Metabolism” among the most significantly enriched categories.

### Natural antisense transcripts, carbon metabolism and nitrogen metabolism are possibly associated

We further examined metabolic and biological pathways that can be affected by natural antisense transcripts (NAT) expression by searching for NATs assigned to KEGG pathways (Kanehisa et al. 2011). NAT expression is common among organisms (Chen et al. 2012a; Lapidot and Pilpel 2006), and these transcripts are able to regulate gene expression through different mechanisms (Britto-Kido et al. 2013; Magistri et al. 2012). In sugarcane, differential expression of NATs involved in sucrose metabolism and photosynthesis was observed under drought stress (Lembke et al. 2012). Sugarcane NATs can also be regulated by the circadian clock independently to their sense counterparts (Hotta et al. 2013).

**Fig. 3:**
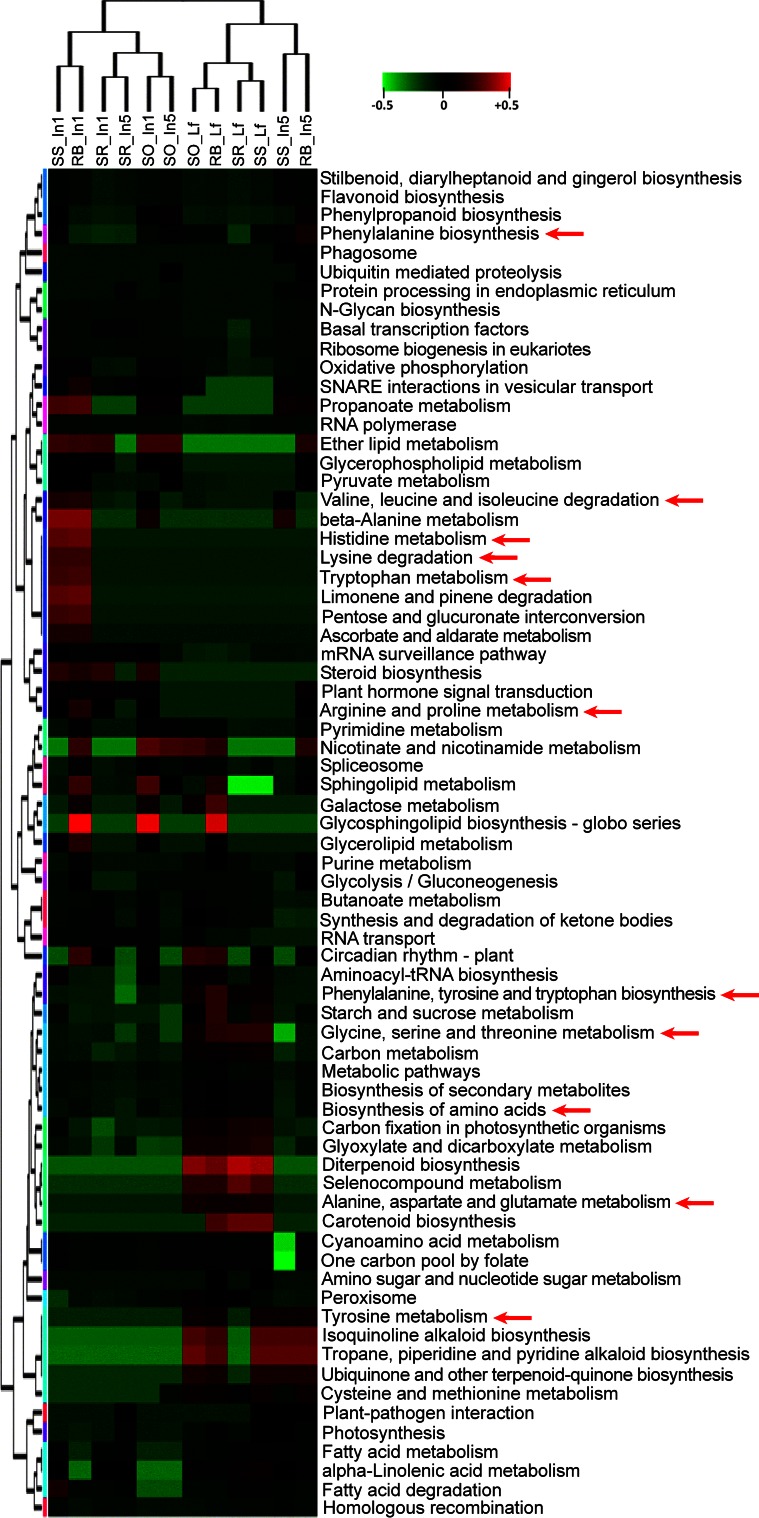
Hierarchical clustering of KEGG pathway activity scores for antisense transcripts. Pathway activity comprises expression data for all enzymes/genes in a given KEGG pathway transformed into a single activity score for each pathway for each sample. The samples are indicated as follows (genotype_tissue): RB, RB867515; SO, *S. officinarum*; SR, *S. robustum*; SS, *S. spontaneum*; In1, immature internodes; In5, intermediate internodes; Lf, leaf. Arrows indicate antisense pathway activity in amino acid-related pathways

The KEGG database has 134 pathways designated to plants. We have identified 71 plant KEGG pathways, out of 134 (53 %), that have at least one expressed NAT mapped to a component gene. In order to select KEGG pathways that were antisense-enriched, we have mapped all SAS against the KEGG database, assigning each SAS to its corresponding KEGG pathway (Nishiyama et al. 2014). Then, we collapsed all expressed antisense probes that complemented the different SAS from a given pathway (i.e., different enzymes in a single pathway) as a single “expression” value, and considered it as a representation of the pathway activity (Fig. 3). Hierarchical clustering of the pathway activity of NATs grouped the samples differently than the global gene expression profiles (Figs. 2, 3). Leaf samples continued to form a single group, however, intermediate internodes of *S. spontaneum* and RB867515 clustered far from the other internode samples and were closer to the leaf samples. Several KEGG pathways related to carbon assimilation and carbohydrate metabolism showed NAT expression, including “Starch and Sucrose Metabolism” and “Carbon Fixation”, in leaves (Fig. 3). Amino acid metabolism was highly regulated by antisense mechanisms in sugarcane, as pathways of 16 out of the 20 protein-forming amino acids could be detected based on NAT expression (Fig. 3), most of which were located in up-regulated gene clusters. As leaf nitrogen is an important feature for biomass accumulation in sugarcane (van Heerden et al. 2010), it is possible that NAT expression affecting amino acid metabolism may underlie the mechanisms that control nitrogen balance and mobilization within plants. However, further validation should be done to prove this hypothesis.

### Differentially expressed genes (DEGs) between genotypes

**Fig. 4:**
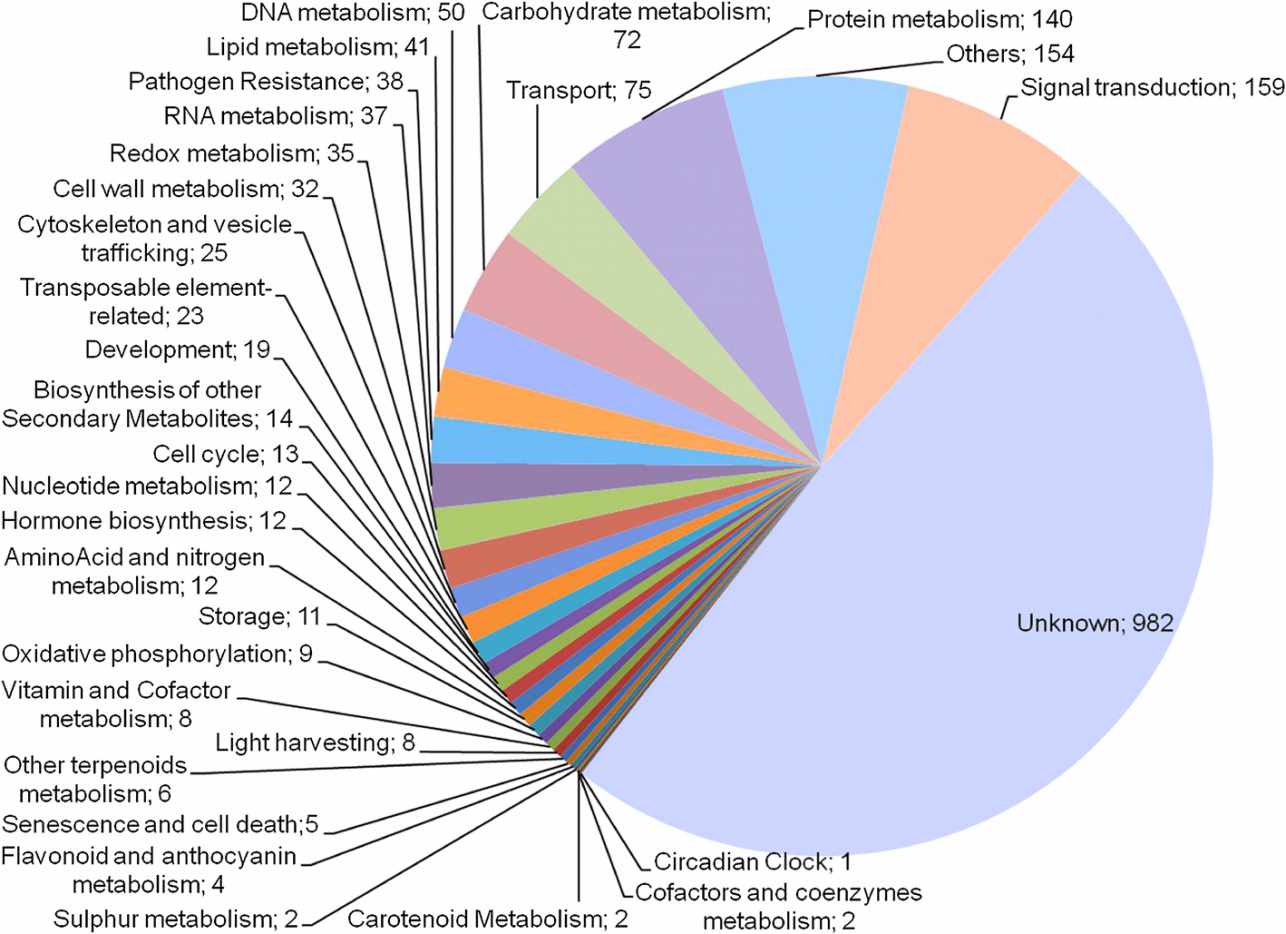
Functional categories of the 2003 differentially expressed SAS in the ancestral genotypes in relation to the commercial variety RB867515 in the three analyzed tissues

**Fig. 5:**
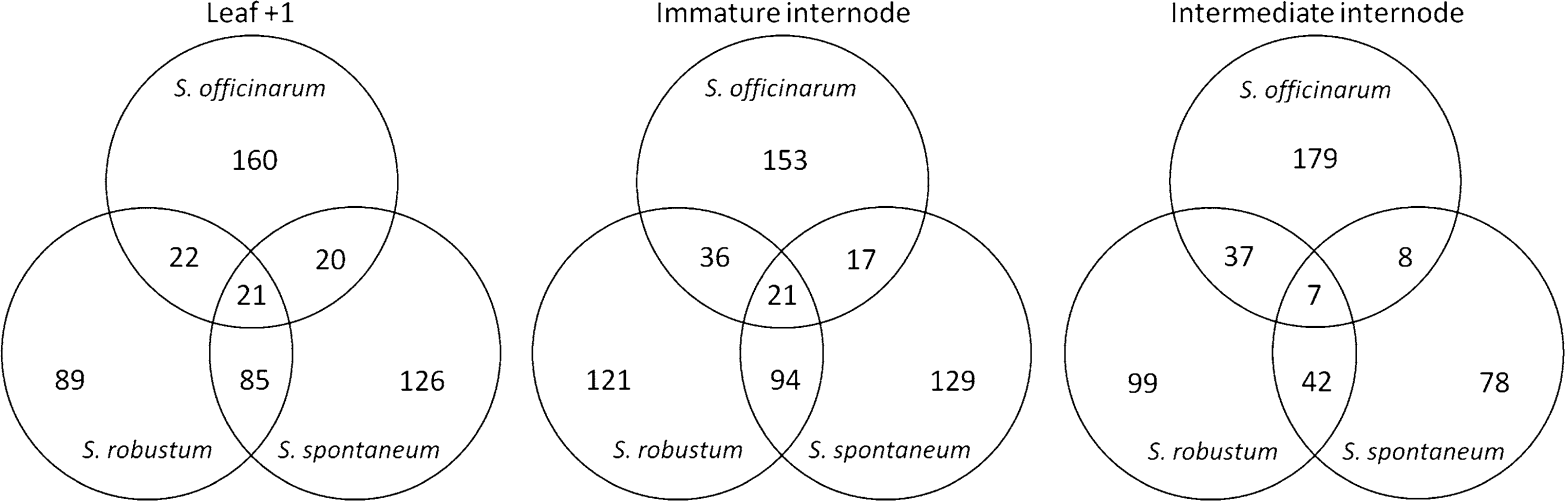
Comparison of differentially expressed genes in each genotype. Number of DEGs in each genotype and shared by different genotypes

Hybridizations were performed with samples from each ancestral genotype against RB867515, such that the commercial variety was used as reference for all comparisons. Genes that were differentially expressed between the ancestral genotypes and RB867515 were identified using HTself (Lembke et al. 2012; Vencio and Koide 2005). We have identified 223, 217 and 252 DEGs in the *S. officinarum*, *S. robustum* and *S. spontaneum* leaf samples (Table 3), respectively. These numbers are similar to those obtained in immature internodes: 227, 272 and 261 DEGs in *S. officinarum*, *S. robustum* and *S. spontaneum*, respectively. However, in intermediate internodes, the numbers of DEGs were lower in *S. robustum* and *S. spontaneum* (185 and 135, respectively) than in *S. officinarum* (231). Interestingly, the number of down-regulated genes was greater than the number of up-regulated genes for all hybridizations (Table 3). In total, 2003 DEGs were identified in all of the experiments (Online Resource 2). The most representative category considering all DEGs was “Signal Transduction” (159), after “Unknown Protein” (Fig. 4). Venn diagrams (Fig. 5) show that most of the DEGs are exclusive for each genotype, and only 7 to 21 genes are differentially expressed in all genotypes for each tissue, which means there is a wide diversity of DEGs. On the other hand, *S. spontaneum* and *S. robustum* share much more DEGs (85, 94 and 42 for leaf, immature and intermediate internodes, respectively) than *S. officinarum* with the two others, which reinforces the pattern observed in Fig. 2, where these two ancestor genotypes are clustered together.

**Table 3:** Experimental design and number of differentially expressed genes in each ancestral genotype in relation to the reference (RB867515) in the three analyzed tissues

Tissue	Hybridization			Number of differentially expressed genes		
	Ancestral	Versus	Reference	Up	down	Total
Leaf+1	*S. officinarum*	Versus	RB867515	71	152	223
	*S. robustum*	Versus	RB867515	100	117	217
	*S. spontaneum*	Versus	RB867515	107	145	252
Immature internodes	*S. officinarum*	Versus	RB867515	89	138	227
	*S. robustum*	Versus	RB867515	104	168	272
	*S. spontaneum*	Versus	RB867515	90	171	261
Intermediate internodes	*S. officinarum*	Versus	RB867515	107	124	231
	*S. robustum*	Versus	RB867515	82	103	185
	*S. spontaneum*	Versus	RB867515	50	85	135

The observed functional category enrichment was different in the ancestral genotypes. *S. robustum* showed “Carbohydrate Metabolism” and “Redox Metabolism” among the most enriched categories, whereas *S. spontaneum* showed “RNA Metabolism” and “Protein Metabolism”. *S. officinarum* displayed “Hormone Biosynthesis” and “Pathogen Resistance” as the most enriched categories, except for the categories with no specified functions (“Unknown” and “Others”) (Table 4). Also, *S. officinarum* presented some categories that were not enriched in both other genotypes, such as “Signal Transduction” and “DNA Metabolism”. Interestingly, in *S. officinarum*, “Flavonoid and Anthocyanin Metabolism” was also among the enriched categories. Altered expression of flavonoid biosynthetic genes has been reported in hybrids compared with their parents (Peng et al. 2014; Shen et al. 2012a), and there is evidence linking flavonoid contents to freezing tolerance in Arabidopsis heterosis (Korn et al. 2008) and to auxin transport (Peer and Murphy 2007). Similarly, as RB867515 is a hybrid derived from *S. officinarum*, it is possible that flavonoid biosynthesis might be related to the superior performance of RB867515 over *S. officinarum*. The precise role of flavonoids in sugarcane heterosis should be further studied.

**Table 4:** Enriched functional categories of differentially expressed genes in each ancestral genotype in relation to the commercial variety RB867515 for the three analyzed tissues

*S. officinarum* versus RB867515		*S. robustum* versus RB867515		*S. spontaneum* versus RB867515	
e-score	Description	e-score	Description	e-score	Description
6.63E−63	Unknown	2.79E−43	Unknown	4.07E−50	Unknown
3.36E−07	Hormone biosynthesis	7.78E−05	Others	2.23E−05	Others
4.30E−06	Pathogen Resistance	6.90E−02	Carbohydrate metabolism	1.43E−02	RNA metabolism
2.10E−05	Others	0.12116	Redox metabolism	1.63E−02	Protein metabolism
7.47E−05	Protein metabolism	0.14272	Protein metabolism	5.68E−02	Lipid metabolism
2.10E−04	Redox metabolism	0.17438	Transport	0.20129	Vitamin and Cofactor metabolism
2.25E−04	Transposable element-related	0.17700	RNA metabolism		
1.09E−03	Signal Transduction	0.18299	Hormone biosynthesis		
9.12E−03	Flavonoid and anthocyanin metabolism	0.20249	Amino acid and nitrogen metabolism		
1.32E−02	Transport				
7.02E−02	DNA metabolism				
0.19963	Amino acid and nitrogen metabolism				

We selected 40 occurrences of DEGs for confirmation via quantitative real-time PCR (qPCR) analysis, achieving 82.5 % validation (Online Resource 3) considering only the direction of the fold change (up or down), but not its magnitude. This percentage is similar to the findings of a study describing the same oligoarray platform (Lembke et al. 2012), and it is in agreement with previous reports showing a high correlation between qPCR and microarray platform results when the direction of gene expression is the main evaluated parameter (Dallas et al. 2005; Morey et al. 2006).

### Sucrose accumulation may be regulated by energy metabolism and sugar transporters

Although the four genotypes can be separated into high and low Brix° plants (Table 1), the expression of genes involved in sucrose biosynthesis and breakdown does not correlate with these differences. Only one major gene involved in sucrose metabolism, sucrose synthase (Table 5), was found to be differentially expressed, but only among high Brix° plants. This poor correlation of gene expression with sucrose accumulation was previously reported in a study on maturing sugarcane stems (Casu et al. 2003). One explanation for this finding is that sucrose metabolism is strongly regulated by post-translational modifications, such as phosphorylation by Snf1-related protein kinases, or SnRKs (Halford and Hey 2009). A SnRK-interacting protein showed differential expression between high and low Brix° plants (Table 5). Moreover, the “Signal Transduction” category, which comprises kinases, phosphatases and transcription factors (TFs), was the second most represented category among the DEGs (Fig. 4), and it was also enriched in the analysis of *S. officinarum* versus RB867515 (Table 4). In addition, four sugar transporters showed a direct correlation with sucrose contents, as two of them were up-regulated in *S. officinarum* and the other two were down-regulated in *S. robustum* and *S. spontaneum* (Table 5). Expression of sugar transporters has been reported to be relatively abundant in sugarcane maturing (Casu et al. 2003). This type of transporter might be responsible for phloem loading, and regulation of the activity and expression of these transporters might contribute to sucrose content by controlling sink-source relationships (Ainsworth and Bush 2011). In fact, manipulation of the gene expression of sugar transporters can increase carbohydrate accumulation in *Verbascum phoeniceum* leaves (Zhang and Turgeon 2009), protein levels in wheat seeds (Weichert et al. 2010), and the cotyledon growth rate in pea (Rosche et al. 2002). Moreover, the two sugar transporters up-regulated in *S. officinarum* belong to the SWEET subfamily of transporters, which it has been shown to participate in phloem loading in Arabidopsis (Chen et al. 2012b). Furthermore, one of the sugar transporters down-regulated in both low Brix° plants, a putative sugar transporter type 2a, has been implicated to phloem loading in sugarcane (Casu et al. 2003). Taken together, these results support the idea that phloem loading by sugar transporters may be a key step for sucrose accumulation in sugarcane.

**Table 5:** List of differentially expressed SAS in the ancestral genotype in relation to the reference (RB867515)

SAS	Functional category	Annotation	*S. officinarum*			*S. robustum*			*S. spontaneum*		
			In1	In5	L	In1	In5	L	In1	In5	L
SCEQLB1065B01.g	Carbohydrate metabolism	Sucrose synthase		↑							
SCACRZ3111E02.g	Carbohydrate metabolism	Putative SnRK1-interacting protein 2				↓	↓		↓	↓	
SCCCLR1079B06.g	Carbohydrate metabolism	Phosphoglycerate kinase	↓	↓		↓					
SCMCRT2089E02.g	Carbohydrate metabolism	Pyruvate decarboxylase	↓	↓							
SCSBFL1105F08.g	Carbohydrate metabolism	6-phosphofructokinase 3							↑		↑
SCSBSB1057D05.g	Carbohydrate metabolism	Starch phosphorylase			↓			↓			
SCUTAM2089E05.g	Carbohydrate metabolism	Beta-amylase	↓		↓		↑	↑			↑
SCJLFL3014G01.g	Oxidative phosphorylation	ATP synthase subunit epsilon							↑	↑	↑
SCCCRT2002G08.g	Transport	Bidirectional sugar transporter SWEET14			↓					↓	
SCEQRT3C03F06.g	Transport	putative hexose carrier protein HEX6			↑						
SCCCRZ2C01E03.g	Transport	Bidirectional sugar transporter SWEET11		↑							
SCSFRT2067F07.g	Transport	Putative sugar transporter type 2a						↓			↓
SCEQRT2091A08.g	Cell wall metabolism	Xyloglucan endotransglycosylase hydrolase (XTH)			↓						
SCJLST1020H07.g	Cell wall metabolism	Xyloglucan endotransglycosylase hydrolase (XTH)		↓							
SCRUFL3067G01.b	Cell wall metabolism	Xyloglucan endotransglycosylase hydrolase (XTH)				↑					
SCCCCL4006H09.g	Cell wall metabolism	Beta-Expansin	↓								
SCCCCL5072C04.g	Cell wall metabolism	Beta-Expansin					↓				
SCCCLB1023H08.g	Cell wall metabolism	Beta-Expansin							↑		
SCCCLR1048B09.g	Cell wall metabolism	Alpha-Expansin		↑			↑				
SCEZLB1008D12.g	Transport	Aquaporin TIP4-2					↓	↓			
SCAGLR2033E03.g	Transport	Aquaporin PIP1-2							↓	↓	↓
SCEQRT2028C04.g	Transport	Aquaporin PIP1-5	↓		↓	↑	↑				
SCQGFL1096G10.g	Transport	Aquaporin PIP2-1			↓						
SCJFRT1060F02.g	DNA metabolism	Histone H3	↓	↓	↓						
SCQGLR1041C10.g	DNA metabolism	Histone H2B	↑	↑	↑						
SCCCRZ2C04H07.g	DNA metabolism	Histone H1				↓			↓		
SCCCLR1C04B01.g	DNA metabolism	Histone H2B				↓	↓	↓	↓	↓	↓
SCCCLR2C02B06.g	DNA metabolism	Histone H2B.1	↑	↑				↓			
SCCCRZ2002A05.g	DNA metabolism	Histone H3							↓		↓
SCCCLR1068F12.g	DNA metabolism	histone H3 (H3-1.1)							↓	↓	↓
SCRFLR2038C05.g	DNA metabolism	Histone H4							↓		↓
SCCCLR2001D01.g	DNA metabolism	Histone H4 variant TH091		↑							
SCCCLR1067B01.g	Signal transduction	LIM-domain binding protein				↓	↓	↓	↓	↓	↓

In1, immature internodes; In5, intermediate internodes; L, leaf+1

This list shows only some DEGs of interest. Complete list of DEGs can be found at Online Resource 2

*Saccharum officinarum* and *S. spontaneum* showed an evident contrast in exhibiting, respectively, down- and up-regulation of genes related to carbohydrate degradation and energy generation, such as phosphofructokinase, phosphoglycerate kinase and an ATP synthase subunit (Table 5). The higher degree of lignification present in *S. spontaneum* demands more energy because each gram of lignin requires 2.6–3.0 g of glucose to be synthesized (Amthor 2003), and this expression profile may suggest that these genes are important for *S. spontaneum* to allocate carbon to lignin biosynthesis, while *S. officinarum* allocates it to sucrose accumulation. Indeed, we observed an increasing in lignin content in *S. spontaneum* along the culm from 12.9 % in immature internodes to 19.3 % in mature internodes (Table 1), whereas no variation was found in *S. officinarum*, approximately 14 % for both immature and mature internodes (Table 1). Moreover, the lower expression of these genes in *S. officinarum* can drive the carbon flux to sucrose accumulation.

Some genes are frequently identified in studies on differential expression in sugarcane. Expansins, XTHs and aquaporins have been shown to present differential expression in sugarcane varieties with contrasting Brix° contents (Papini-Terzi et al. 2009) and in response to drought stress (Lembke et al. 2012). Expansins and XTHs have also been found to be differentially expressed in different internodes of the same plant (Casu et al. 2007). Expansins and XTHs act breaking hydrogen bonds between cellulose microfibrils (McQueen-Mason and Cosgrove 1994) and, latter, remodeling cell wall polysaccharides (Buckeridge 2010; Eklof and Brumer 2010), facilitating cell growth. Aquaporins are also involved in cell expansion (Chen et al. 2013). It has been speculated that changes in cell expansion might lead to increased sucrose accumulation capacity (Papini-Terzi et al. 2009). Our results also revealed several representatives of these three protein groups that were differentially expressed (Table 5), which may suggest that the involvement of these proteins in different pathways is a common feature in sugarcane genotypes (Casu et al. 2007; Lembke et al. 2012; Papini-Terzi et al. 2009).

Several histones showed lower transcription levels in the ancestral genotypes compared to RB867515, especially in low Brix° plants (Table 5). Genomic stress arising from interspecific hybridization may result in up-regulation of epigenetic control through histone modifications to select which homologous genes will be expressed (Chen and Tian 2007; Hu et al. 2013). As RB867515 is an interspecific hybrid, it is reasonable to hypothesize that the observed up-regulation of histones may be related to higher turnover of these proteins or even to recycling of histones subjected to irreversible modifications, such as histone tail-clipping (Santos-Rosa et al. 2008), which can be involved in certain types of epigenetic control. Furthermore, we identified a gene from the LIM domain-binding protein family that was exclusively down-regulated in all tissues in low Brix° plants (Table 5). A LIM domain-containing protein was reported to be involved in the regulation of histone expression (Moes et al. 2013). It is possible that epigenetic control plays an important role in sugarcane heterosis, explaining the better performance of hybrids compared with their ancestors, as observed for other species (Chen 2013). Still, the hypothesis of an association of histone differential expression to epigenetic control needs additional validation.

Histone differential expression among genotypes may also be a consequence of hybrid higher growth, where the cells in the internodes might be in S-phase, requiring more histones to be synthesized for nucleossome formation in the new cells, and the differential expression of expansins, XTHs and aquaporins (genes likely to be involved in cell growth and expansion) supports this idea. This should be particularly true for immature internodes, which is a tissue near to apical meristematic and under intense cell divisions. However, in intermediate internodes cell proliferation is much lower than compared to immature internodes, and cell expansion and elongation are the main processes taking place in this tissue. In fact, a recent work shows that in intermediate internodes of RB867515, parenchyma cells have higher diameter than cells in the same tissue of *S. spontaneum* (Guzzo de Carli Poelking et al. 2015), suggesting a higher degree of cell expansion in the hybrid, which are also in agreement of differential expression of expansins, XTHs, and aquaporins. Still, this down regulation of histones in low Brixº plants needs to be further investigated to confirm any hypothesis.

### Screening for sugarcane TFs involved in cell wall metabolism via co-expression network analysis

**Fig. 6:**
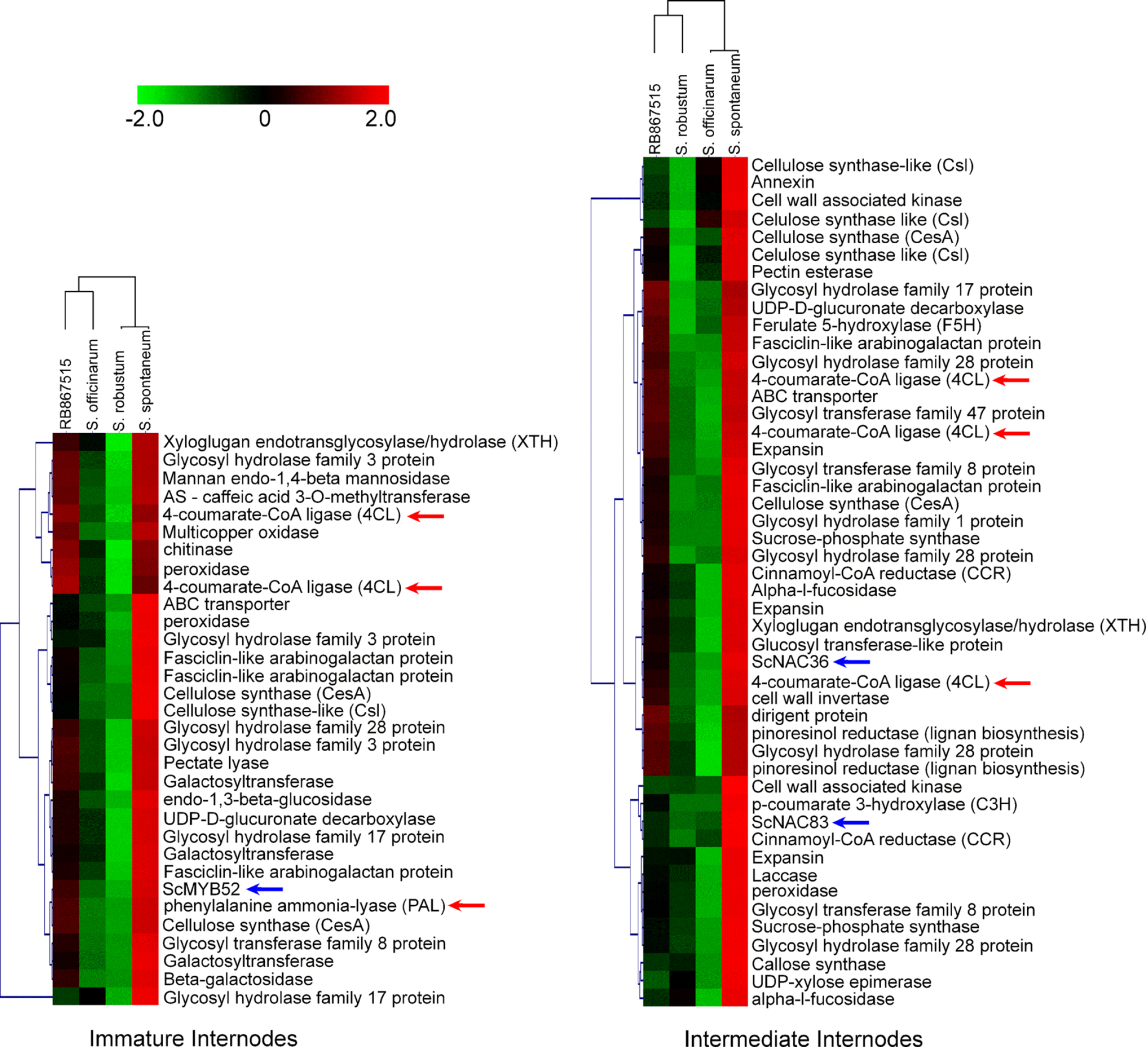
Hierarchical clusters of cell wall-related genes that are up-regulated in *S. spontaneum* in immature and intermediate internodes using normalized log2 expression data (signal intensity analysis). *Red arrows* highlight the lignin biosynthetic genes *pal* and *4* *cl*, and blue arrows highlight NAC and MYB transcription factors

To develop tailor-made biomass for different applications, a strong knowledge of sugarcane cell wall metabolism and regulation is needed. Lignin is a hydrophobic polymer crosslinked to hemicellulose, conferring strength and rigidity to the cell wall (Boerjan et al. 2003; Carpita 1996). Lignin is one of the main substances responsible for biomass recalcitrance, as it prevents hydrolytic enzymes from reaching polysaccharides, and its degradation products inhibit hydrolytic enzymes and fermentation (Keating et al. 2006). On the other hand, lignin exhibits a high combustion energy (Raveendran and Ganesh 1996; White 1987), which is important for biomass burning to produce electricity, and it can be used as raw material to produce several high value chemicals, such as DMSO and vanillin (Calvo-Flores and Dobado 2010). Therefore, understanding and achieving control of lignification and lignin structure/composition is expected to be of great interest (Sticklen 2008). Despite all the information produced in plant models, specially Arabidopsis and poplar (Boerjan et al. 2003; Bonawitz and Chapple 2010; Zhong and Ye 2009), only a few systematic studies have been carried out to address lignin metabolism in sugarcane (Bottcher et al. 2013; Guzzo de Carli Poelking et al. 2015; Vicentini et al. 2015). In our results, despite the observed differences in growth and lignin contents (Table 1; Fig. 1), cell wall-related genes were not abundant in the DEGs between genotypes (only 32 genes, Fig. 4). This result might be due the high stringency of the HTself method, and we therefore applied a different approach to identify DEGs that might be directly correlated with phenotypic differences. Here, using expression data on cell wall-related genes from internodes (see methods) we carried out a hierarchical clustering analysis to identify genes whose expression was clearly correlated with the distinct lignin deposition pattern observed in *S. spontaneum* (Table 1; Fig. 1). Similar to the HTself method, our signal intensity-based analysis followed the expression pattern observed in qPCR assays (Online Resource 4). Genes involved in lignin biosynthesis, especially *phenylalanine ammonia*-*lyase* (*pal*) and *4*-*coumarate:CoA ligase* (*4cl*), fell into clusters in which gene expression was higher in *S. spontaneum* (Fig. 6), correlating with its higher lignin content. For an independent validation, we employed a different set of plants of the same genotypes to investigate the gene expression of several genes involved in lignin biosynthesis via qPCR. We verified the up-regulation of the biosynthetic genes *pal* and *4cl*, as well as *caffeic acid 3*-*O methyltransferase* (*comt*) and *ferulate 5*-*hydroxylase* (*f5h*) in the intermediate internodes of *S. spontaneum* (Online Resource 5), confirming the above mentioned results. These findings are similar to those previously reported for hybrid sugarcane genotypes with contrasting lignin contents (Bottcher et al. 2013). These authors conducted a qPCR analysis of several genes involved in lignin biosynthesis and suggested that the correlation between gene expression and lignin deposition is not always direct, although some genes showed expression correlated with lignin contents, including *pal* and *4cl*. Taken together, these results may suggest that these two genes are key players in lignin biosynthesis in sugarcane, and may be good targets for future analyses, including genetic manipulations. We also identified two TFs from the NAC family (*ScNAC36* and *ScNAC83*) and one from the MYB family (*ScMYB52*), two families that contain members which are key regulators of cell wall metabolism (Zhong et al. 2007a, b, 2008, 2010; Zhong and Ye 2012), showing expression correlated with lignin contents (Fig. 6), suggesting that these TFs are good candidates for further analysis.

**Fig. 7:**
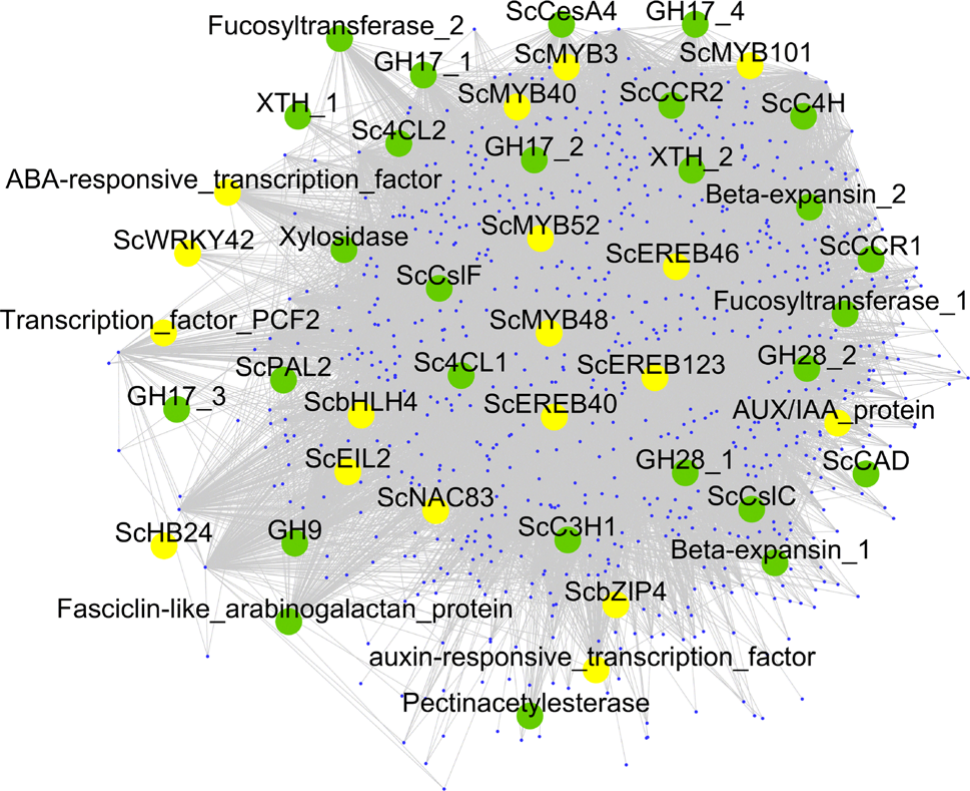
Co-expression network of sugarcane generated using the lignin biosynthetic genes *pal* and *4* *cl* as guides. The network comprises 736 genes (nodes); *green* and *yellow* nodes represent cell wall-related genes and transcription factors, respectively, and all other genes are depicted as small blue nodes. Cell wall-related genes (*green nodes*) are shown as follows: lignin biosynthetic genes [phenylalanine ammonia lyase (ScPAL2); 4-coumatare-CoA ligase (Sc4CL1-2); cinnamate 4-hydroxylase (ScC4H); p-coumarate 3-hydroxylase (ScC3H1); cinnamoyl-CoA reductase (ScCCR1-2); cinnamyl alcohol dehydrogenase (ScCAD)]; carbohydrate-related genes [cellulose synthase (ScCesA4); cellulose synthase-like (ScCslC and F); glycosyl hydrolase (GH) families 3 (xylosidase), 9 (beta-1,4-glucanase), 17 (beta-1,3-glucanase) and 28 (polygalacturonase); fucosyltransferase; pectinacetylesterase; xyloglucan endotransglycosylase/hydrolase (XTH)]; cell wall proteins: (beta-expansins; Fasciclin-like arabinogalactan protein). Yellow nodes indicate the following transcription factors: MYB (ScMYB3, 40, 48, 52, 101); NAC (ScNAC83); WRKY (ScWRKY42); AP2-EREBP (ScEREB40, 46, 123); bHLH (ScbHLH4); bZIP (ScbZIP4); EIL (ScEIL2); Homeobox (ScHB24); AUX/IAA protein; PCF2; ABA-responsive transcription factor; auxin-responsive transcription factor. The nomenclature of the lignin biosynthetic genes and transcription factors is based on (Bottcher et al. 2013) and (Yilmaz et al. 2009), respectively

In an attempt to data mine TFs that are potentially involved in cell wall biosynthesis in sugarcane, we built a co-expression network using the R-package WGCNA (Langfelder and Horvath 2008) employing expression data from internodes (Fig. 7), since we have identified differences in lignin content in the culm among genotypes, especially intermediate internodes (Table 1). We selected two genes from lignin biosynthesis pathway as bait, *4cl* and *pal,* as mentioned above, as their expression is correlated with the higher lignification observed in *S. spontaneum*. The network comprises 736 genes (nodes) and the picture (Fig. 7) highlights the two categories of genes which are the main focus of this work: cell wall-related genes (green nodes) and transcription factors (yellow nodes). Functional category enrichment analysis (Table 6) shows that this network (Fig. 7) is enriched in genes related to cell wall metabolism. Several TF families have been reported to include members controlling cell wall biosynthesis, especially NAC and MYB (Zhong et al. 2007a, b, 2008, 2010; Zhong and Ye 2012), but also the AP2-EREBP (Ambavaram et al. 2011), homeobox (Li et al. 2012) and WRKY families (Wang et al. 2010; Yu et al. 2013). Here, we found 18 TFs in the sugarcane co-expression network belonging to these families (Fig. 7; Table 7). Except for the gene *f5h* (Zhao et al. 2010), lignin biosynthesis is known to be directly regulated by MYB TFs (Fornale et al. 2010; Ma et al. 2011; McCarthy et al. 2009; Shen et al. 2012b; Sonbol et al. 2009; Zhong et al. 2007a; Zhou et al. 2009), which may explain the high number of MYBs in this network (5/18), as we used lignin biosynthetic genes to guide TF identification in the network. Importantly, two of these TFs, *ScNAC83* and *ScMYB52*, were identified as being up-regulated in *S. spontaneum* (high lignin content) in the signal intensity analysis (Fig. 6).

**Table 6:** Functional category enrichment of network module containing the target gene *Sc4CL* and *pal* (Fig. 7)

e-score	Description
5.5086E−04	Cell wall metabolism
6.4277E−03	Signal Transduction
1.4537E−02	Transport
1.3255E−01	Unknown
5.7145E−01	Others
9.7865E−01	Lipid metabolism

**Table 7:** Transcription factors (TFs) identified in the co-expression network and number of cell wall-related cis-elements (SNBE and SMRE) present in candidate promoter sequences (2.0 kb upstream from 5′ UTR) of each TFs

SAS	Annotation	SMRE^a^	SNBE^b^
SCCCCL4001A01.g	ScWRKY42	4	4
SCCCLR1022B07.g	ABA-responsive transcription factor	3	5
SCCCRZ1004H12.g	ScEIL2	5	6
SCEPRZ1011C11.g	AUX/IAA protein	1	0
SCEPRZ3046G08.g	auxin-responsive transcription factor	1	1
SCJFRT2059H08.g	ScMYB40	9	8
SCRLRZ3041C03.g	ScMYB101	0	6
SCQGST1032D08.g	ScMYB52	0	5
SCQSHR1023B08.g	ScEREB46	1	10
SCMCLR1122H05.g	ScEREB40	0	1
SCEPRZ3129A06.g	ScEREB123	nd^c^	nd
SCCCLR1076F07.g	ScbHLH4	nd	nd
SCCCCL4005C09.g	ScbZIP4	nd	nd
SCEQRT2095E01.g	ScHB24	nd	nd
SCACST3159E04.g	ScMYB3	nd	nd
SCMCRT2105A02.g	ScMYB48	nd	nd
SCCCCL4014A04.g	ScNAC83	nd	nd
SCRLFL4029G02.g	Transcription factor PCF2	nd	nd

All TFs found in the network (Fig. 7) were analyzed

^a^SMREs can appear randomly in the genome every 2.0 kb (Zhong and Ye 2012)

^b^SNBEs can appear randomly in the genome every 1.8 kb (Zhong et al. 2010)

^c^Nd, non-determined (not present in BACs database)

Functional characterization of genes is a time consuming and complicated task, making it necessary to conduct accurate identification of targets. This is especially important for sugarcane, as the production of transgenic plants faces constraints such as low transformation efficiencies, transgene inactivation, and somaclonal variation (Hotta et al. 2010). The investigation of co-expression networks has been demonstrated to be a good approach for identifying candidate genes of interest in several groups of species, such as mammals, insects, yeast and plants (Lee et al. 2004; Movahedi et al. 2012; Ruprecht et al. 2011; Ruprecht and Persson 2012; Stuart et al. 2003; Wang et al. 2012). The identified candidate genes include a new enzyme involved in lignin biosynthesis in Arabidopsis, caffeoyl shikimate esterase (Vanholme et al. 2013); a laccase gene (*SofLAC*) associated with the lignification process in sugarcane (Cesarino et al. 2013); and TFs related to cell wall biosynthesis. A recent study identified over 100 rice TFs in a secondary cell wall co-expression network analysis, several of which clustered into clades containing Arabidopsis cell wall-associated genes in a phylogenetic tree (Hirano et al. 2013a). Transgenic rice plants that contained silencing and overexpression constructs for six of these TFs showed phenotypes related to changes in the cell wall and altered expression of a lignin biosynthetic gene (Hirano et al. 2013b). Following the same approach, we expected to identify sugarcane TFs that also would be good candidates for further analysis. Phylogenetic analysis grouped *ScMYB48, ScMYB3* and *ScMYB52* into a cluster containing the rice TFs *OsMYB64* (Os05g0140100), *OsMYB93* (Os08g0151300) and *OsMYB14* (Os01g0702700) (Online Resource 6), which are components of a rice secondary cell wall co-expression network, and the last TF is closely related to the Arabidopsis secondary cell wall activating TF, *AtMYB46* (Hirano et al. 2013a). Similar to the work of Hirano and colleagues (Hirano et al. 2013a), we identified TF families other than NAC and MYB in our co-expression network, including members of the WRKY, AP2-EREBP, bHLH, AUX/IAA, Homeobox and bZIP families (Fig. 7; Table 7), as well as other non-TF genes, such as beta-1,4-glucanases (GH9), cellulose synthase-like family F (CslF) and xyloglucan endotransglucosylase/hydrolase (XTH) (Fig. 7). Comparative co-expression analysis is a powerful tool for studying gene functions across species (Movahedi et al. 2012), and these genes identified in both sugarcane (this study) and rice (Hirano et al. 2013a) may represent conserved modules in grass cell wall metabolism.

### “In silico” promoter analysis of co-expressed genes shows the presence of binding sites of cell wall related TFs

Cell wall-related TFs act in several layers of control. Generally, TFs from the NAC family, which are usually referred to as master switches, play a primary role by activating downstream TFs, especially from the MYB family, which can in turn activate the transcription of downstream TFs and cell wall enzymes (Ko et al. 2009; McCarthy et al. 2009; Ohashi-Ito et al. 2010; Yamaguchi et al. 2011; Zhong et al. 2008, 2010; Zhong and Ye 2012). Here, the promoter sequences of the TFs in the network were investigated to obtain evidence that they could be direct targets of upstream cell wall-related TFs by searching for regulatory DNA motifs to which secondary cell wall-related TFs would bind. NAC TFs bind to a 19 bp imperfect palindromic sequence ([T/A]NN[C/T][T/C/G]TNNNNNNNA[A/C]GN[A/C/T][A/T]) element known as the secondary wall NAC binding element (SNBE) (Zhong et al. 2010), whereas MYB TFs recognize a 7 bp sequence (ACC[A/T]A[A/C][T/C]) known as the secondary wall MYB responsive element (SMRE) (Zhong and Ye 2012). Using sugarcane BAC genomic sequences (De Setta et al. 2014), we found candidate promoter sequences for 10 out of 18 TFs in the network, 7 of which presented cis-elements in their promoters at a frequency greater than randomly expected (Table 7), including 10 SNBEs in the promoter sequence of *ScEREB46* and 5 SNBEs in the *ScMYB52* promoter. *ScMYB52* and *ScMYB101* only showed SNBEs, suggesting that they may be direct targets of secondary cell wall-related NACs. As mentioned above, *ScMYB52* is up-regulated in *S. spontaneum* (Fig. 6) and is closely related to *OsMYB14*, which, in turn, was identified in a rice secondary cell wall co-expression analysis and is closely related to a key cell wall activating gene *AtMYB46* in Arabidopsis (Hirano et al. 2013a). All of these data may suggest that *ScMYB52* is candidate for being involved in cell wall biosynthesis in sugarcane.

Approximately 25–30 TFs regulating cell wall metabolism have been characterized in the plant model *Arabidopsis* [reviewed by (Hussey et al. 2013)], while considerably fewer TFs have been studied in grasses (Fornale et al. 2010; Hirano et al. 2013b; Ma et al. 2011; Shen et al. 2012b; Sonbol et al. 2009; Valdivia et al. 2013; Yoshida et al. 2013; Yu et al. 2013; Zhong et al. 2011), and none have been investigated in sugarcane. However, grasses exhibit different cell wall compositions and structures [reviewed by (Carpita and McCann 2008; Vogel 2008)], which may imply the existence of grass-specific genes. In fact, there is evidence that the expanded grass clade of MYB TFs, with no putative orthologues in dicot species, may include members involved in secondary cell wall regulation (Zhao and Bartley 2014). Therefore, it is important to obtain a better understanding of cell wall biosynthesis to improve grasses used for biomass production. The results presented here represent a step forward in this context, as we gathered evidence to support candidate TFs for further analyses.

## Conclusion

In this work, we have linked gene expression to biomass phenotypes by comparative expression profiling of three sugarcane ancestral genotypes and one commercial variety, to identify targets for sugarcane biomass accumulation, and to obtain insight regarding the regulatory networks controlling traits of interest, such as cell wall accumulation. We observed genotype-specific expression patterns and a clear distinction of *S. spontaneum* from the other examined plants. Our results suggest that sucrose accumulation in sugarcane may be regulated by other mechanisms than the regulation of the expression of sucrose metabolizing enzymes, including an unexpected differential expression of histones, which may suggest epigenetic regulation of this trait. Additionally, all four genotypes show antisense expression, and it appears that NATs are abundant in genes related to amino acid metabolism. Finally, co-expression network analysis identified a number of TF targets expected to be key regulators of cell wall metabolism in sugarcane, *ScMYB52* being a good candidate for further investigations. In conclusion, our results provide information on gene functions and promoters that have potential for to be used to produce transgenic plants with improved biomass quality and yields.

## Electronic supplementary material

Below is the link to the electronic supplementary material.

Supplementary material 1 (DOCX 13 kb)

Supplementary material 2 (XLSX 1256 kb)

Supplementary material 3 (DOCX 3095 kb)

Supplementary material 4 (DOCX 5002 kb)

Supplementary material 5 (DOCX 3547 kb)

Supplementary material 6 (DOCX 279 kb)
