# Supplementary material for: Co-expression network analysis reveals transcription factors associated to cell wall biosynthesis in sugarcane
Source: Plant Mol Biol. 2016 Jan 28;91:15–35. doi: 10.1007/s11103-016-0434-2 (PMC4837222; doi:10.1007/s11103-016-0434-2)
Supplement: Supplementary file 1 — Supplementary material 1 (DOCX 13 kb) [file 11103_2016_434_MOESM1_ESM.docx]

| Online Resource 1. Enriched functional categories for all expressed genes in each genotype for the three analyzed tissues | | | | | | | | | | |
| --- | --- | --- | --- | --- | --- | --- | --- | --- | --- | --- |
| **RB867515** | |  | ***S. officinarum*** | |  | ***S. robustum*** | |  | ***S. spontaneum*** | |
| **e-score** | **Description** |  | **e-score** | **Description** |  | **e-score** | **Description** |  | **e-score** | **Description** |
| 0 | Oxidative phosphorylation |  | 0 | Oxidative phosphorylation |  | 0 | Oxidative phosphorylation |  | 0 | Oxidative phosphorylation |
| 0 | DNA metabolism |  | 0 | Protein metabolism |  | 0 | RNA metabolism |  | 0 | Light harvesting |
| 0 | Protein metabolism |  | 0 | RNA metabolism |  | 0 | Light harvesting |  | 0 | Cytoskeleton and vesicle trafficking |
| 0 | Cytoskeleton and vesicle trafficking |  | 0 | Cytoskeleton and vesicle trafficking |  | 0 | Cytoskeleton and vesicle trafficking |  | 0 | Flavonoid and anthocyanin metabolism |
| 0 | Light harvesting |  | 0 | Light harvesting |  | 0 | Flavonoid and anthocyanin metabolism |  | 9.1E-17 | Protein metabolism |
| 0 | Nucleotide metabolism |  | 0 | Circadian Clock |  | 3.2E-19 | Protein metabolism |  | 5.4E-15 | Others |
| 0 | Cell cycle |  | 0 | Carotenoid Metabolism |  | 7.9E-12 | Unknown |  | 4.0E-13 | Unknown |
| 0 | Carotenoid Metabolism |  | 5.7E-20 | Unknown |  | 1.8E-10 | Others |  | 1.5E-12 | Signal Transduction |
| 0 | RNA metabolism |  | 1.0E-08 | Signal Transduction |  | 1.5E-09 | Signal Transduction |  | 6.2E-11 | RNA metabolism |
| 0 | Circadian Clock |  | 1.3E-08 | DNA metabolism |  | 1.5E-08 | Transport |  | 1.7E-09 | Transport |
| 0 | Flavonoid and anthocyanin metabolism |  | 3.3E-08 | Others |  | 3.6E-07 | DNA metabolism |  | 1.2E-05 | DNA metabolism |
| 3.6E-51 | Unknown |  | 4.3E-07 | Transport |  | 8.5E-04 | Carbohydrate metabolism |  | 2.1E-04 | Cell cycle |
| 7.8E-19 | Others |  | 2.4E-03 | Cell cycle |  | 3.5E-03 | Cell cycle |  | 4.0E-04 | AminoAcid and nitrogen metabolism |
| 2.0E-18 | Signal Transduction |  | 2.0E-02 | Carbohydrate metabolism |  | 9.2E-03 | AminoAcid and nitrogen metabolism |  | 4.2E-03 | Carbohydrate metabolism |
| 2.0E-11 | Transport |  | 4.1E-02 | AminoAcid and nitrogen metabolism |  | 9.9E-02 | Nucleotide metabolism |  | 6.9E-01 | Nucleotide metabolism |
| 8.9E-05 | Carbohydrate metabolism |  | 6.3E-01 | Lipid metabolism |  | 1.7E-01 | Lipid metabolism |  | 6.9E-01 | Lipid metabolism |
