# Supplementary material for: Co-expression network analysis reveals transcription factors associated to cell wall biosynthesis in sugarcane
Source: Plant Mol Biol. 2016 Jan 28;91:15–35. doi: 10.1007/s11103-016-0434-2 (PMC4837222; doi:10.1007/s11103-016-0434-2)

Online Resource 3. qPCR validation in a third biological replicate of differentially expressed genes identified by microarray analyses. Reference signal is the expression in RB867515. Green and red rectangles below de gene name indicate the expression profile of the ancestor genotype (up or down) in relation to the reference (RB867515), as follows: SO, *S. officinarum*; SR, *S. robustum*; SS, *S. spontaneum*. Symbols denote the experimental point on which the qPCR result confirm (✓) or not (X) the microarray result. Differences in expression were evaluated by t-test (p<0.05) for each pair (ancestor genotype VS RB867515). GeNorm results are shown as M-value (average expression stability) graph, V-value (pairwise variation) graph and thresholds are shown as a green line in each graph. GeNorm interpretation for each tissue analyzed is shown as well. Six endogenous controls were tested to identify the best ones for data normalization. Error bar= SEM; N=3. A, qRT-PCR results and geNorm analysis for immature internodes; B, qRT-PCR results and geNorm analysis for intermediate internodes; C, qRT-PCR and geNorm analysis results for Leaf+1.

**A**


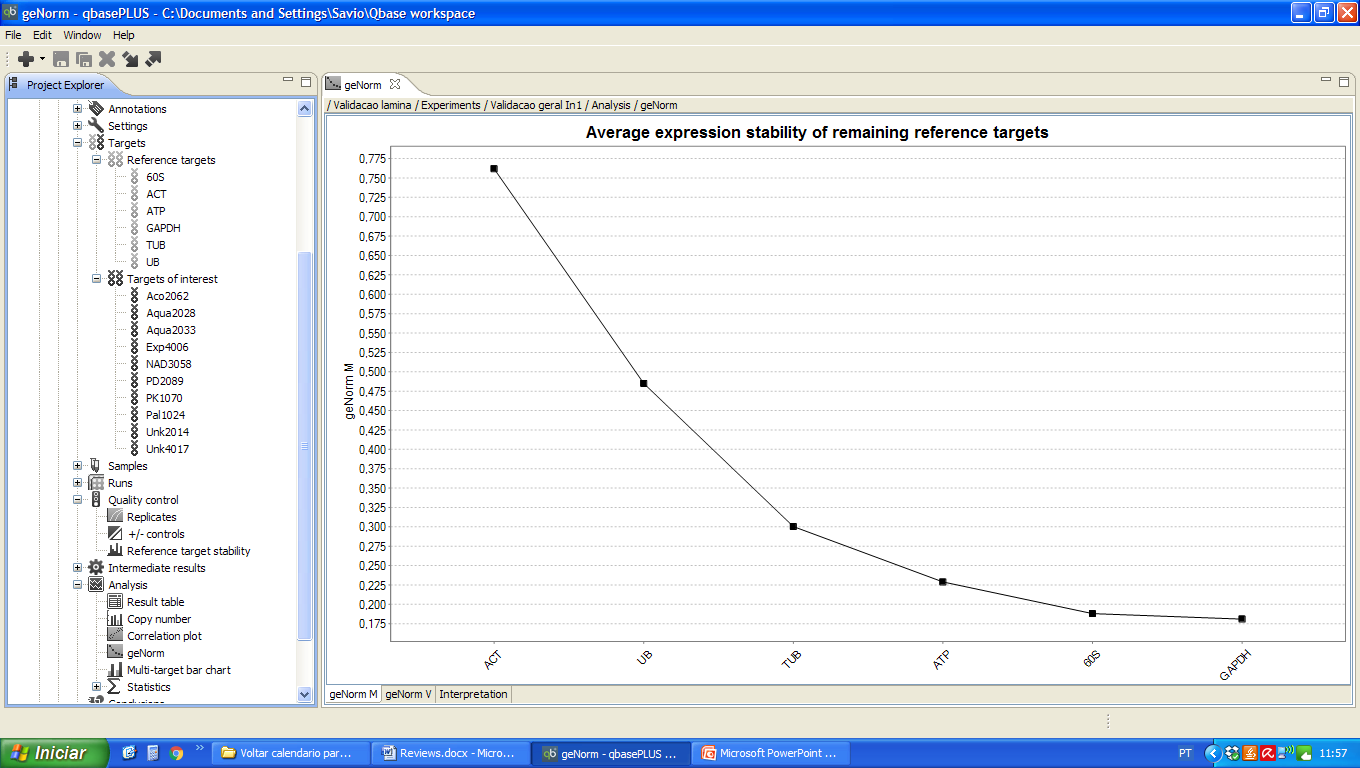

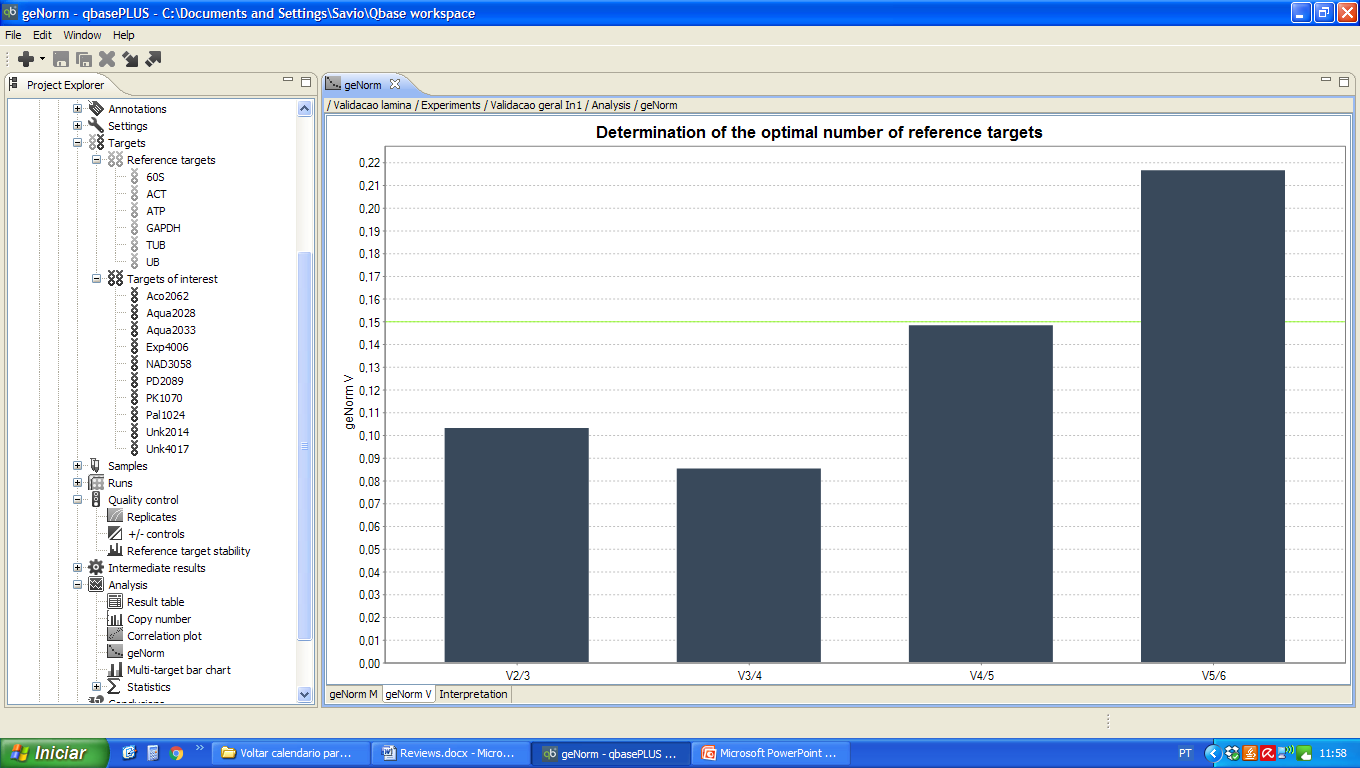

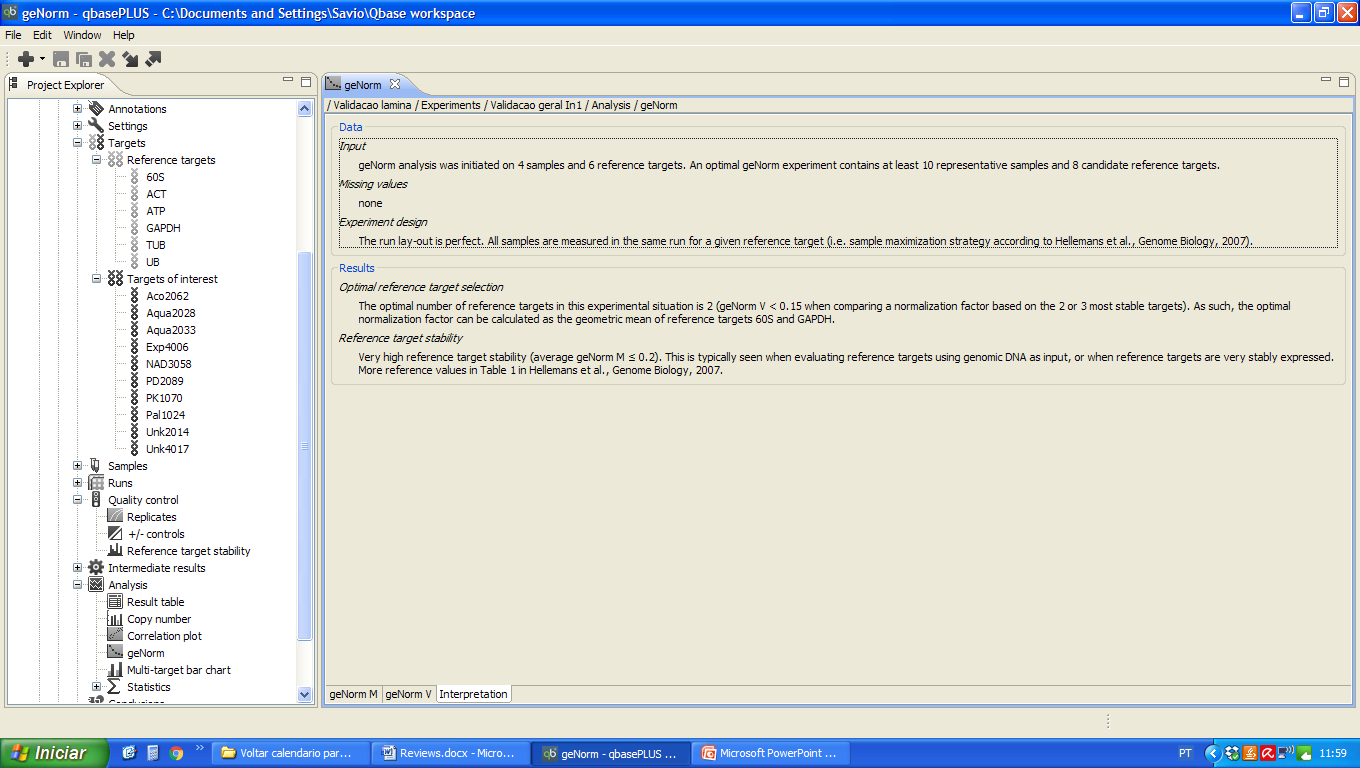


**B**

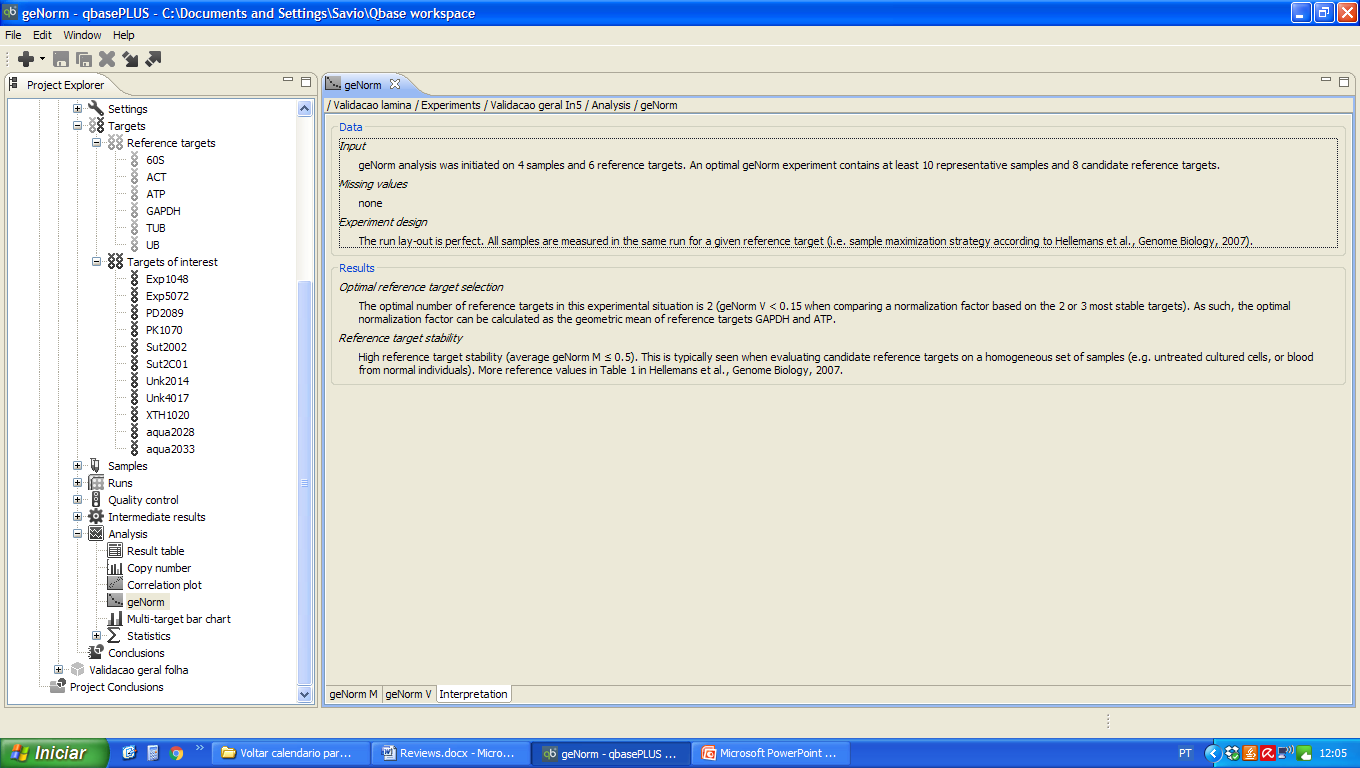

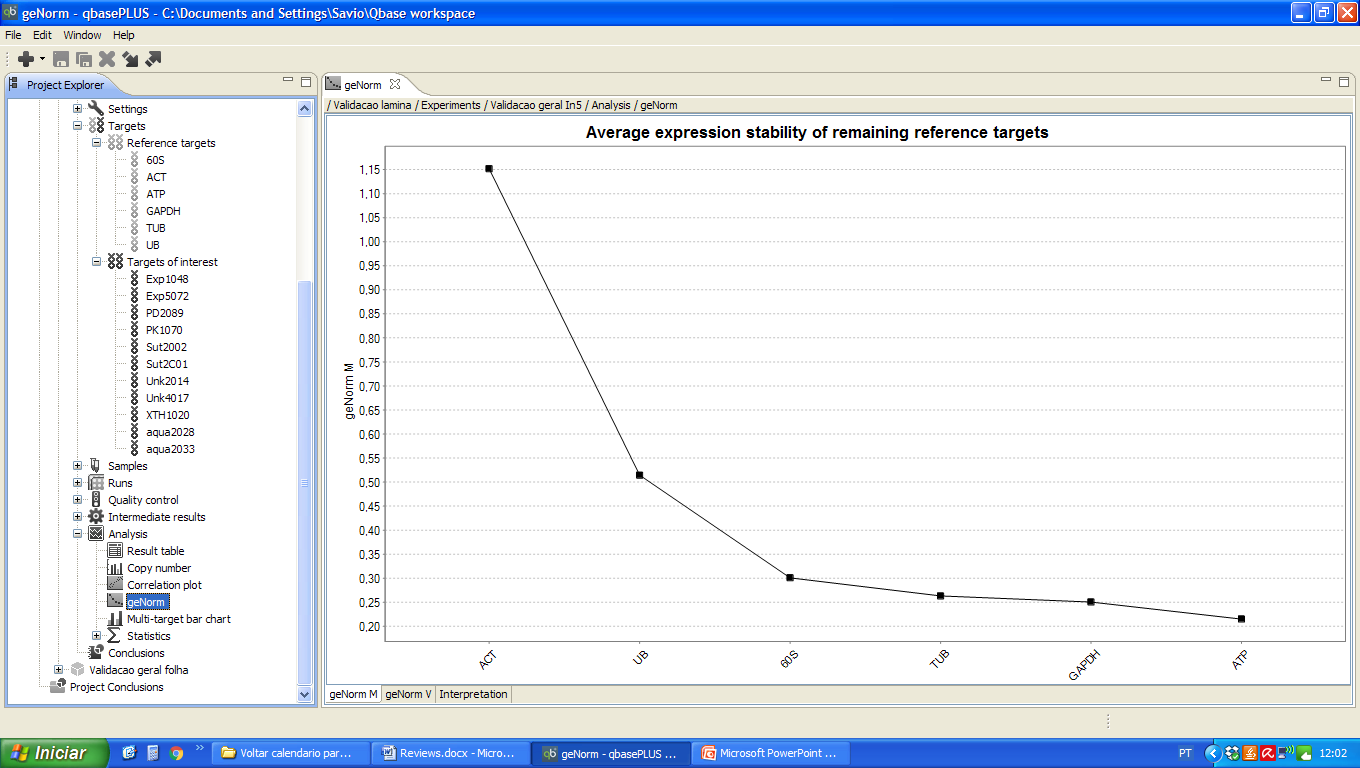

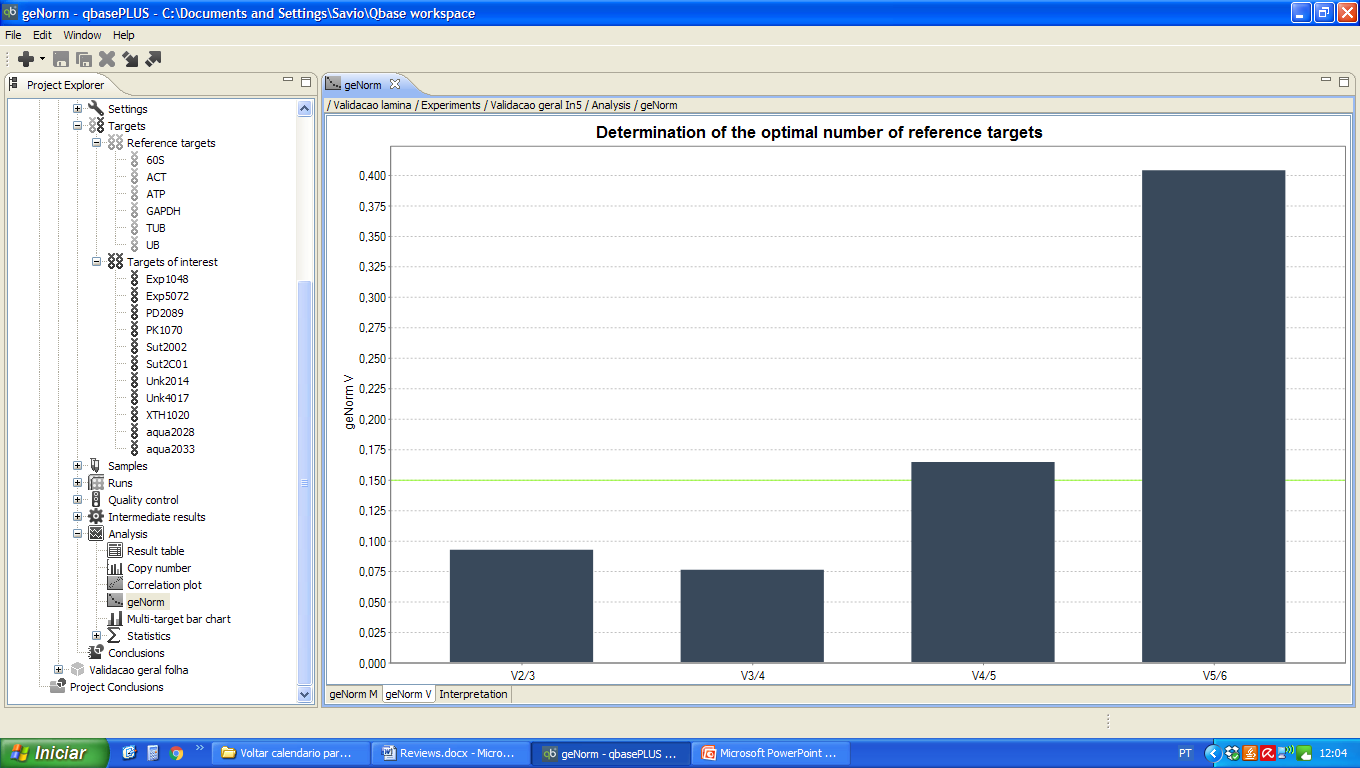


**C**

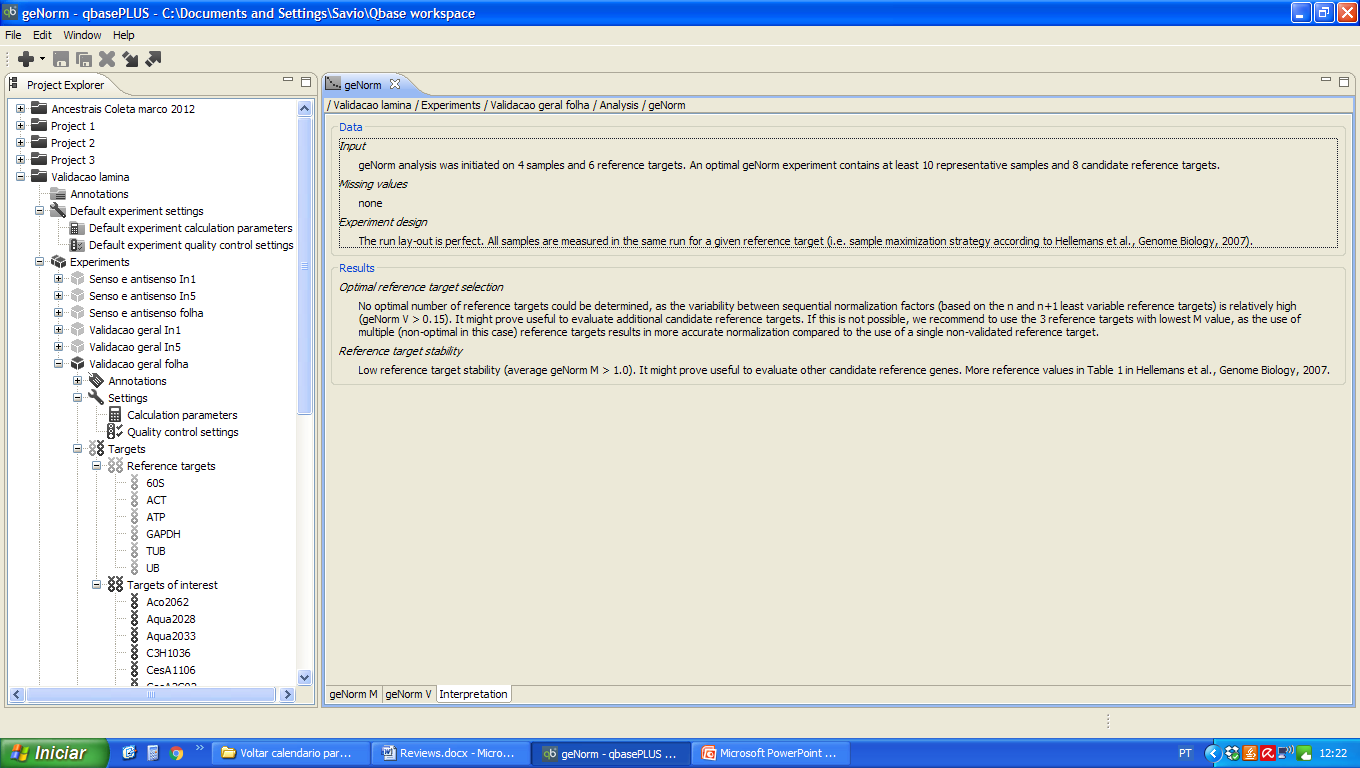

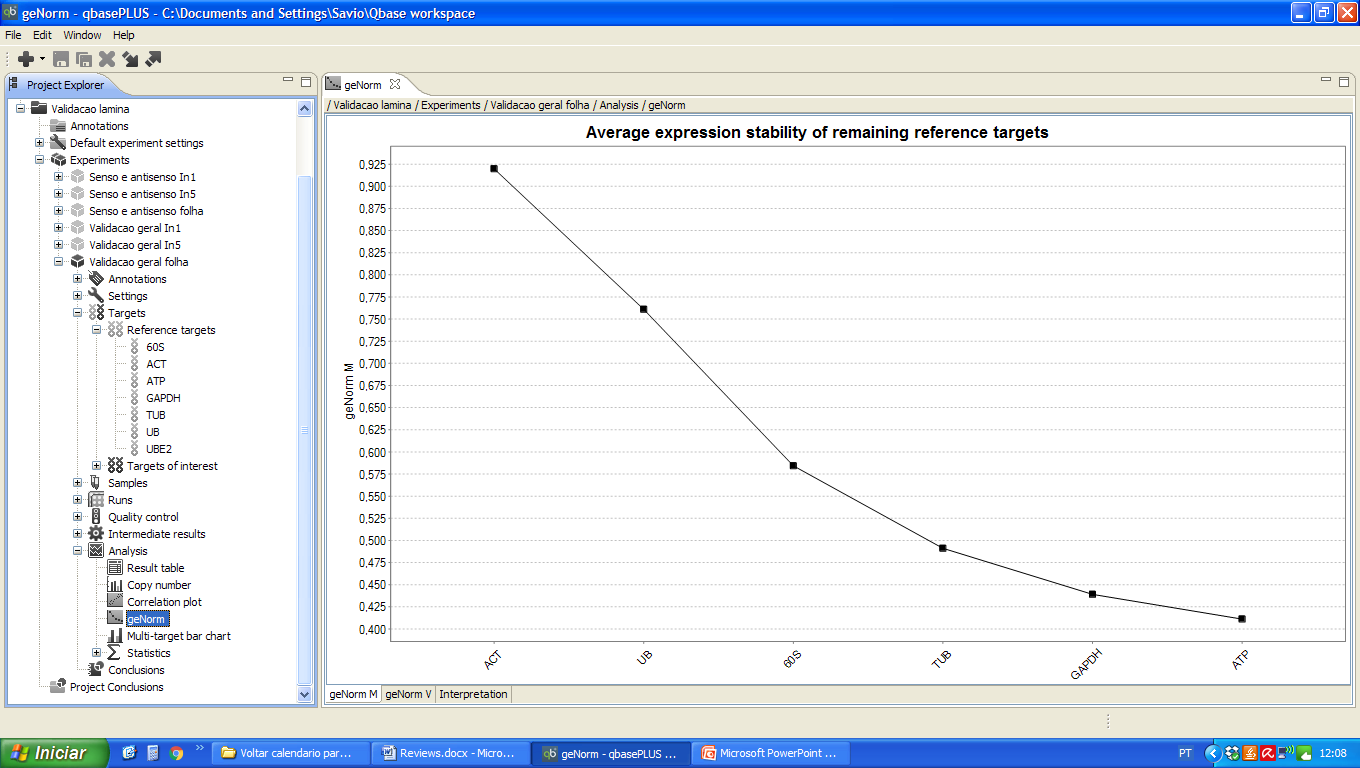

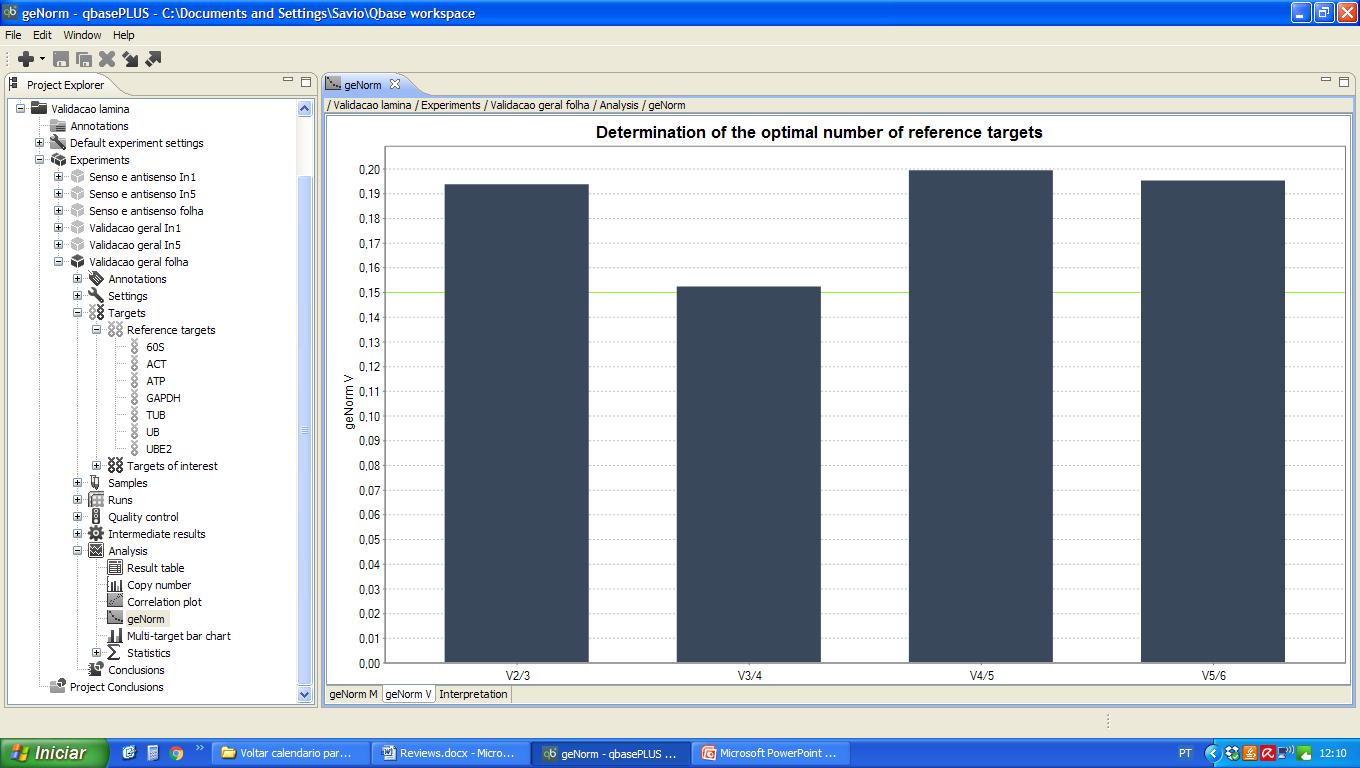

Supplement: Supplementary file 3 — Supplementary material 3 (DOCX 3095 kb) [file 11103_2016_434_MOESM3_ESM.docx]
