# Supplementary material for: Co-expression network analysis reveals transcription factors associated to cell wall biosynthesis in sugarcane
Source: Plant Mol Biol. 2016 Jan 28;91:15–35. doi: 10.1007/s11103-016-0434-2 (PMC4837222; doi:10.1007/s11103-016-0434-2)

**Online Resource 4. qPCR validation of the signal intensity analysis.** The graphs show three examples of gene expression compared between qPCR assays (bars, left axis) and the signal intensity in microarrays (lines, right axis) for immature (A) and intermediate (B) internodes. The signal intensity-based analysis (lines) followed the expression pattern observed in the qPCR assays (bars). For example, the expression of the SAS SCQGST1032D08.g (MYB) in immature internodes (A, third graph) is lower in *S. officinarum* than in RB867515 and *S. spontaneum* in both qPCR and microarray analyses; the expression of the SAS SCCCCL4007E12.g (UDP-D-glucuronate decarboxylase) in intermediate internodes (B, first graph) is similar in *S. officinarum* and in *S. robustum* and these two show lower expression than in RB867515 and *S. spontaneum*. C and D show GeNorm results, for immature and intermediate internodes, respectively, as M-value (average expression stability) graph, V-value (pairwise variation) graph and thresholds are shown as a green line in each graph. GeNorm interpretation for each tissue analyzed is shown as well. Six endogenous controls were tested to identify the best ones for data normalization.


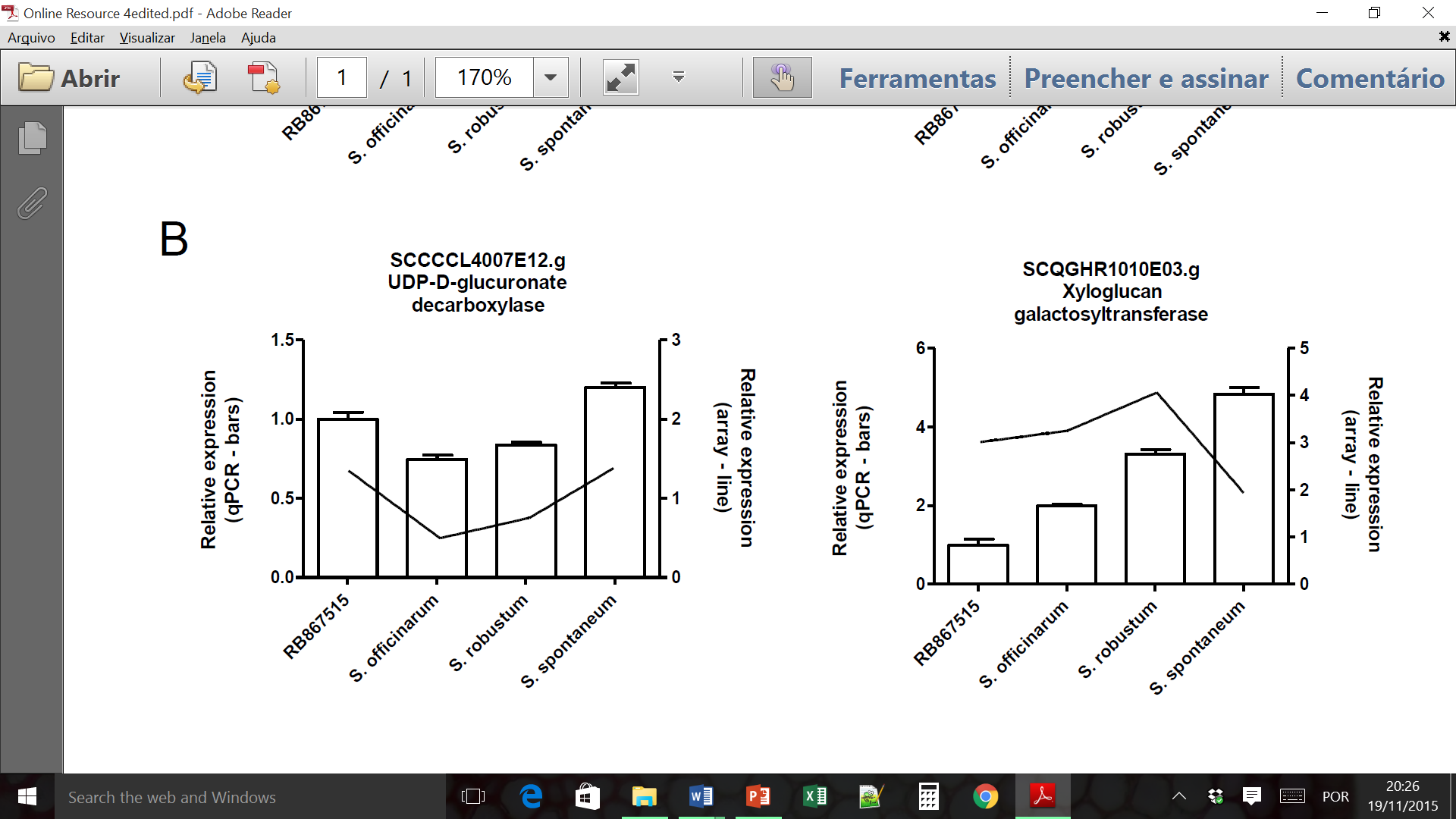

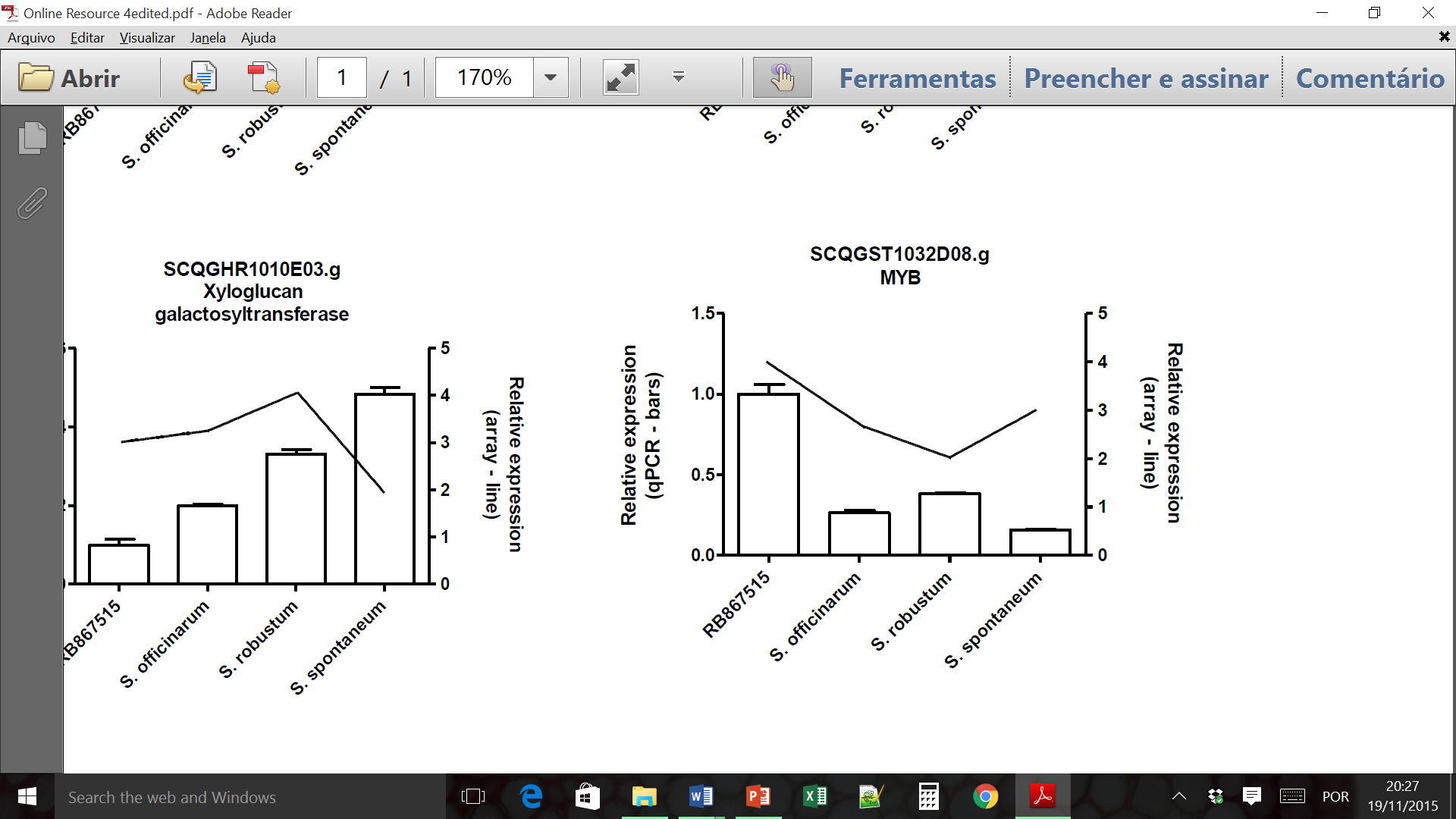

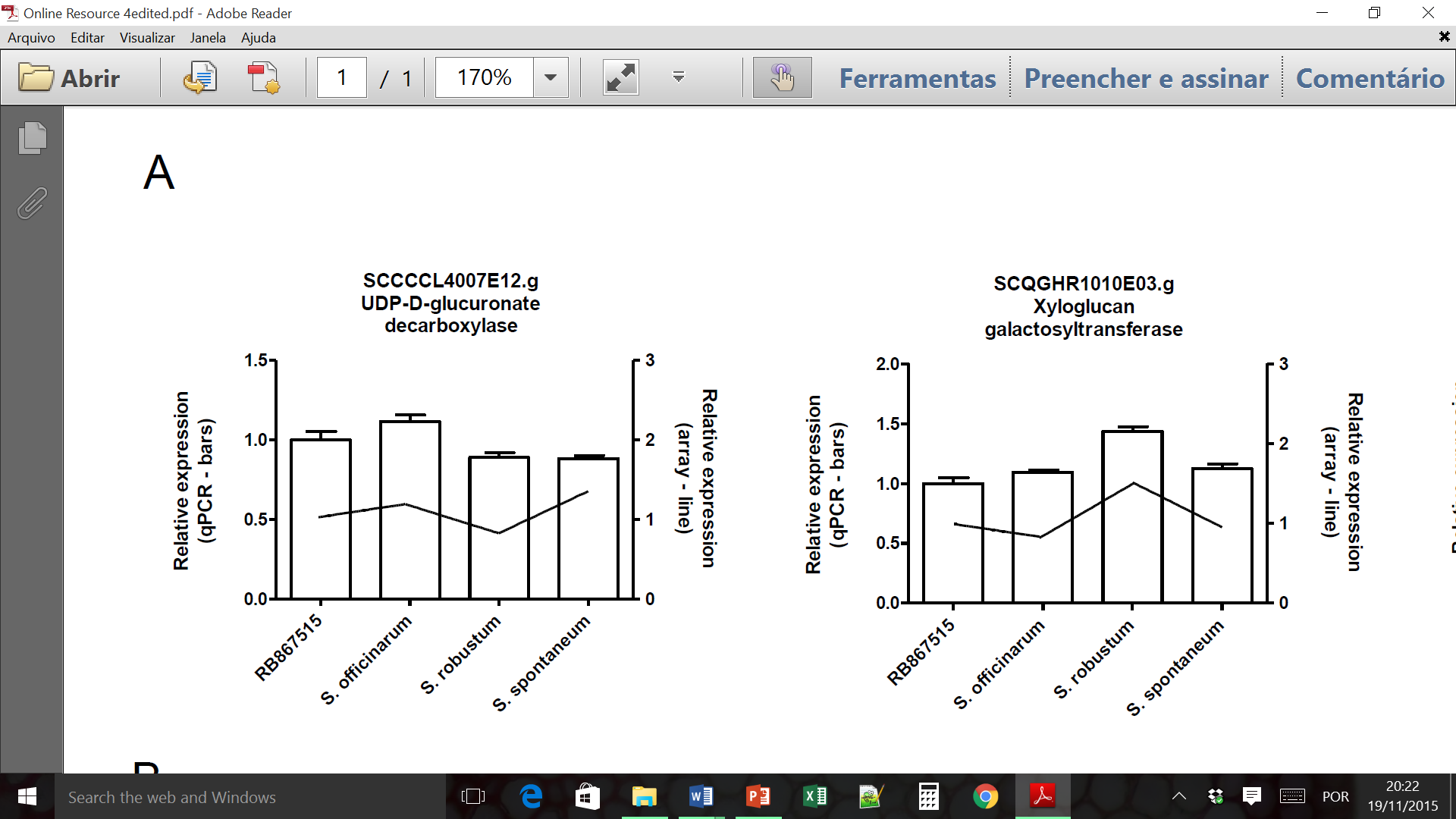

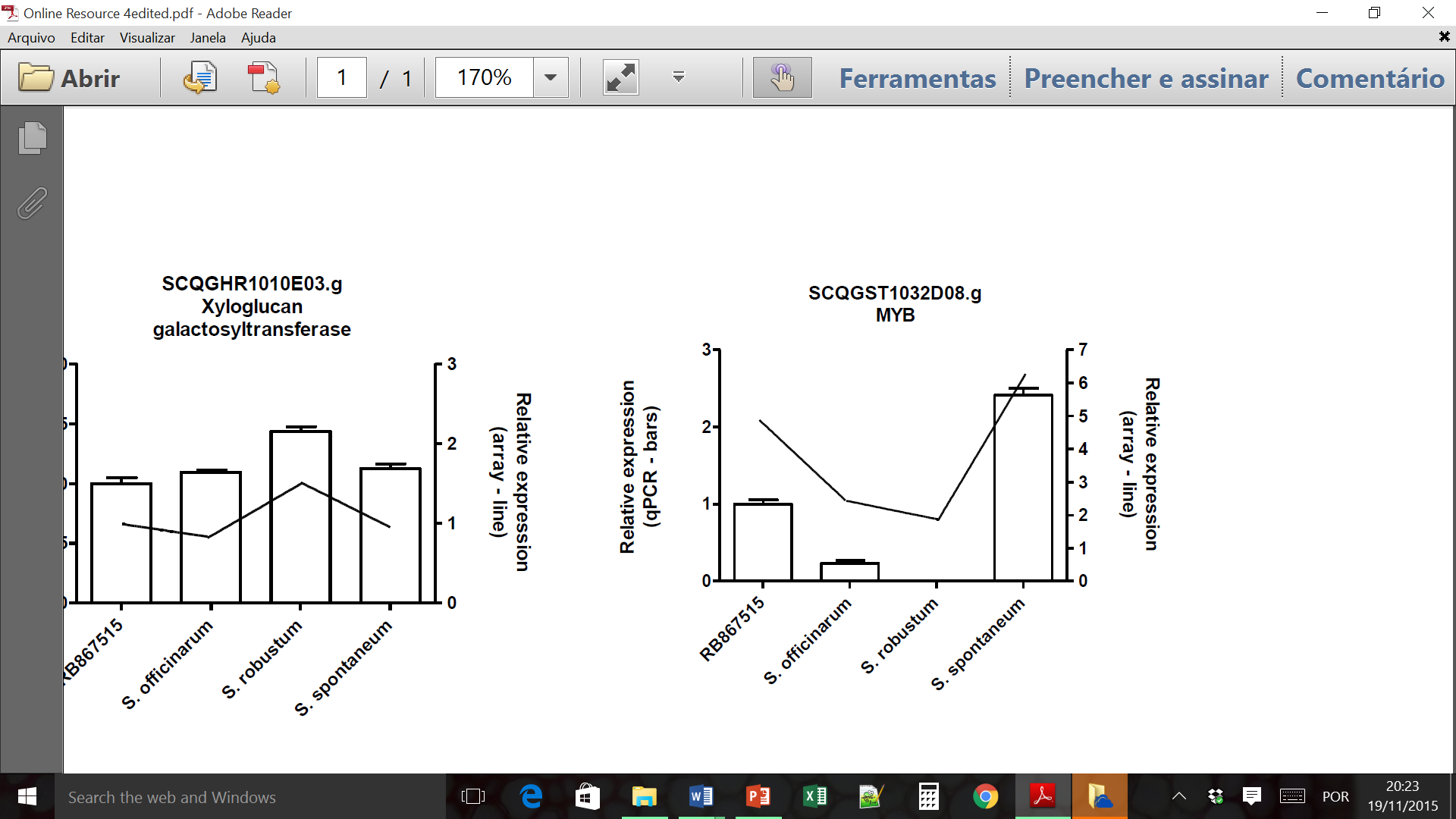


**C**


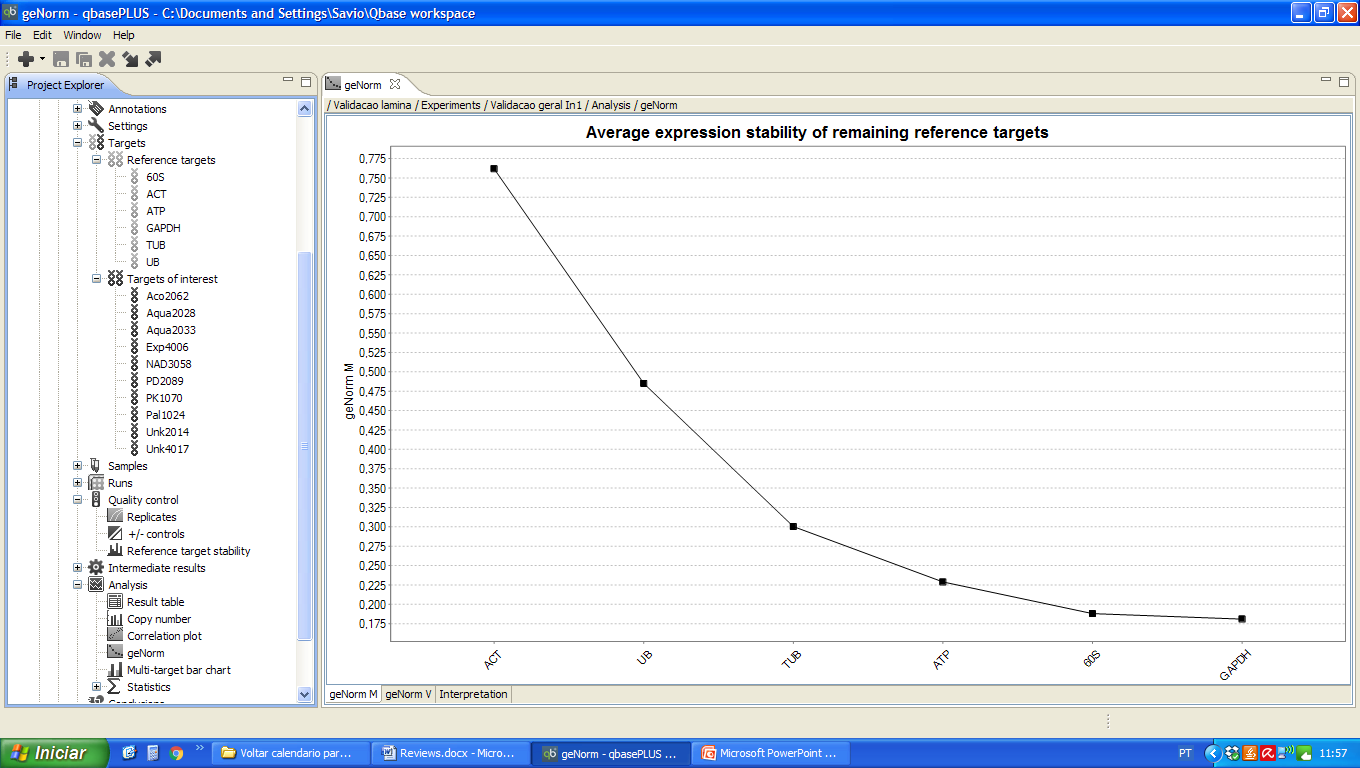

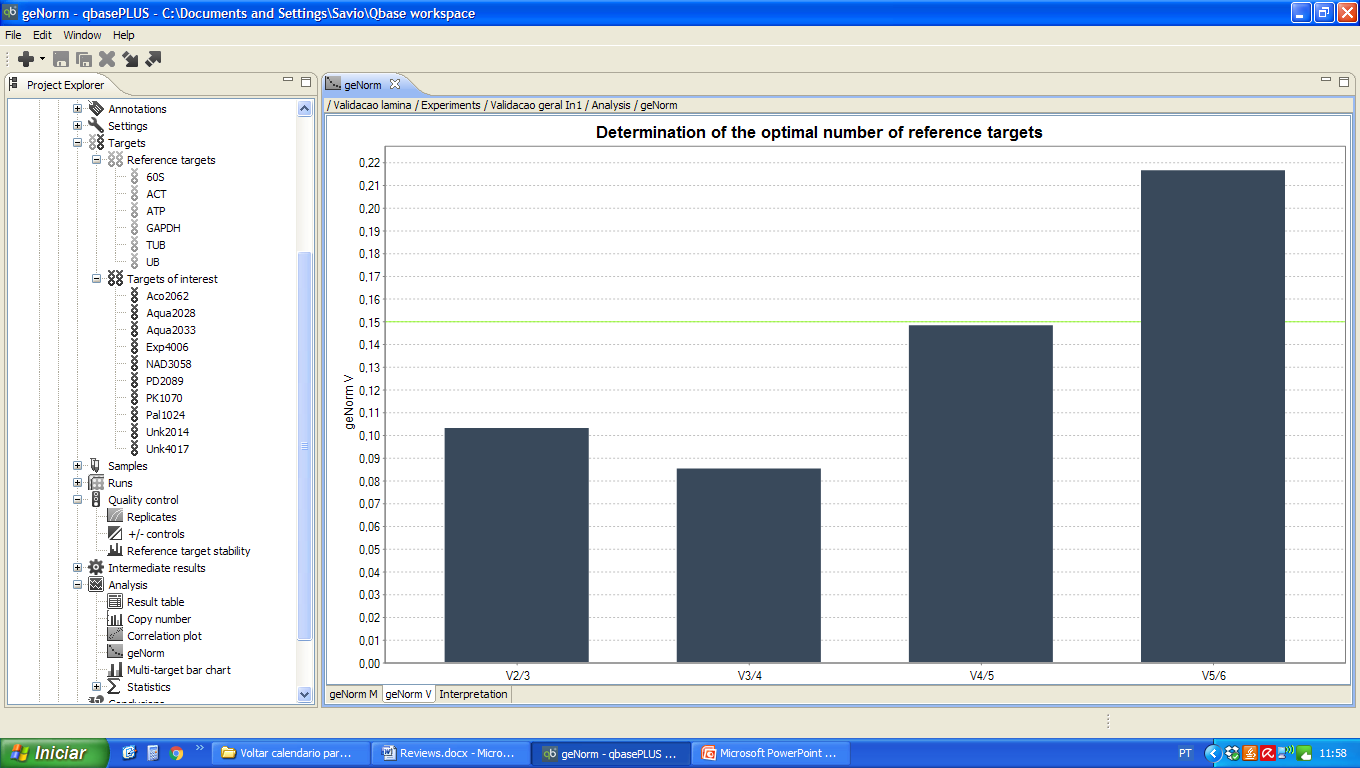

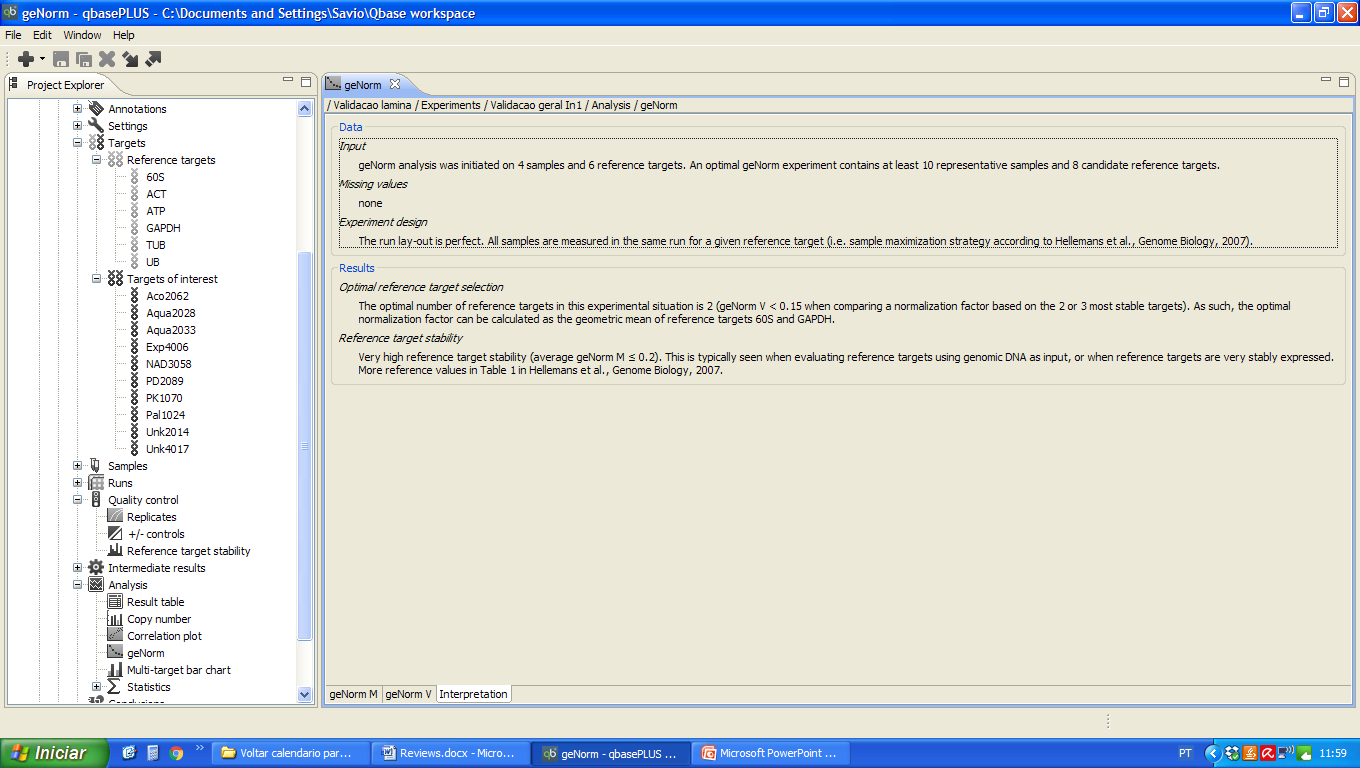


**D**


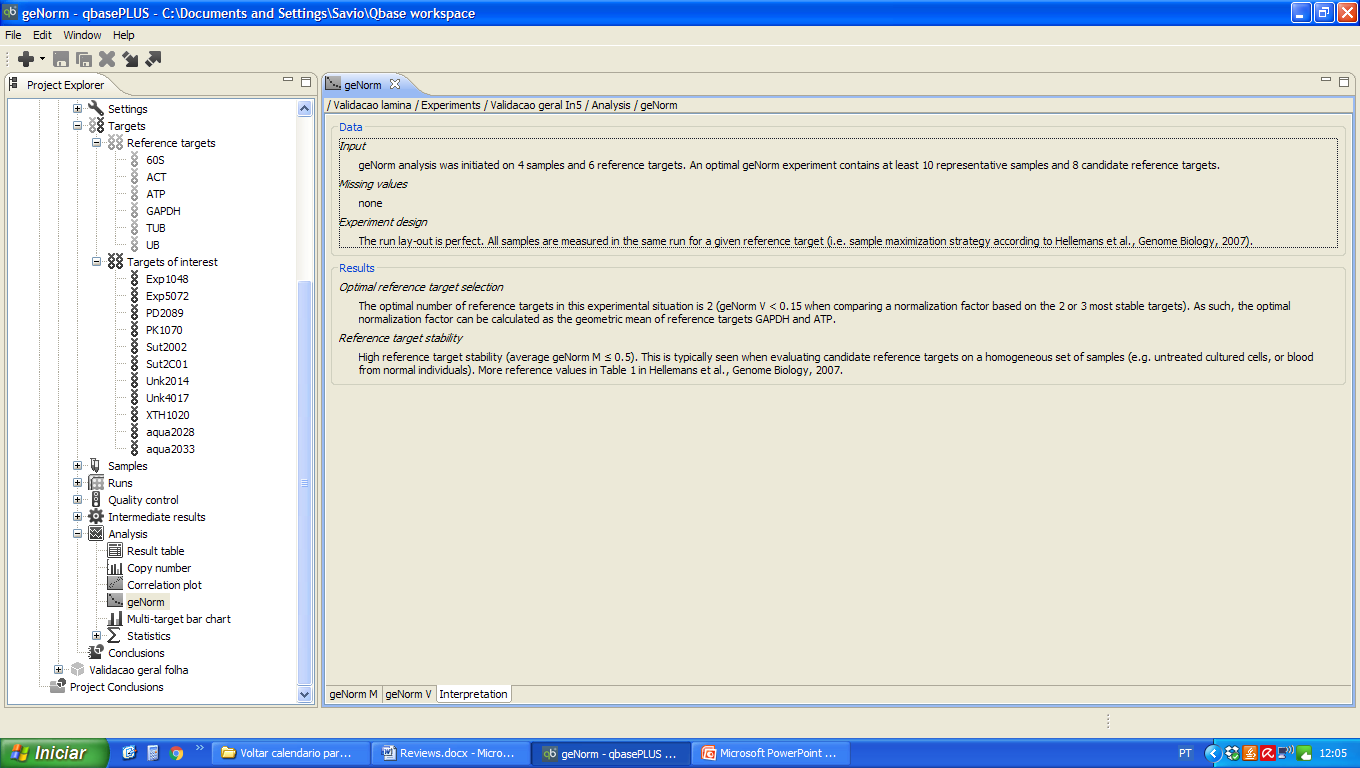

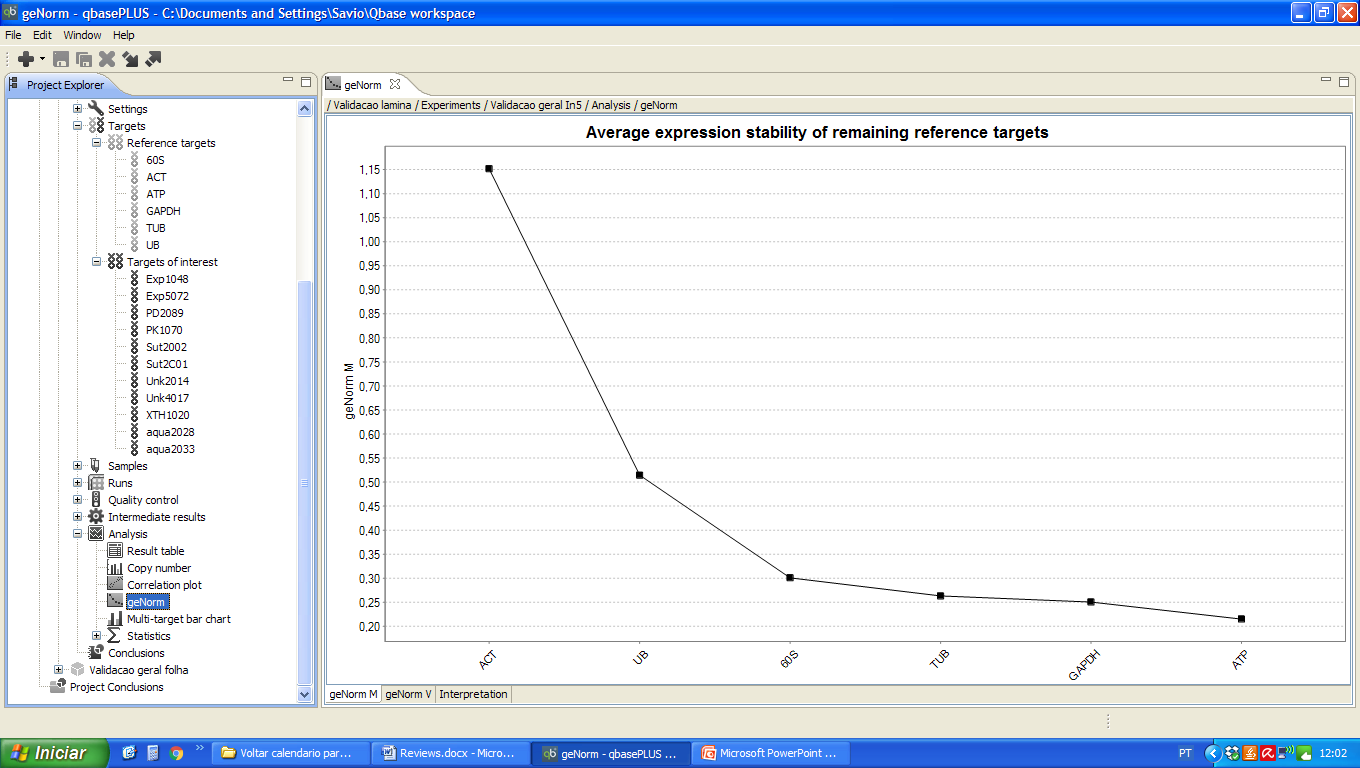

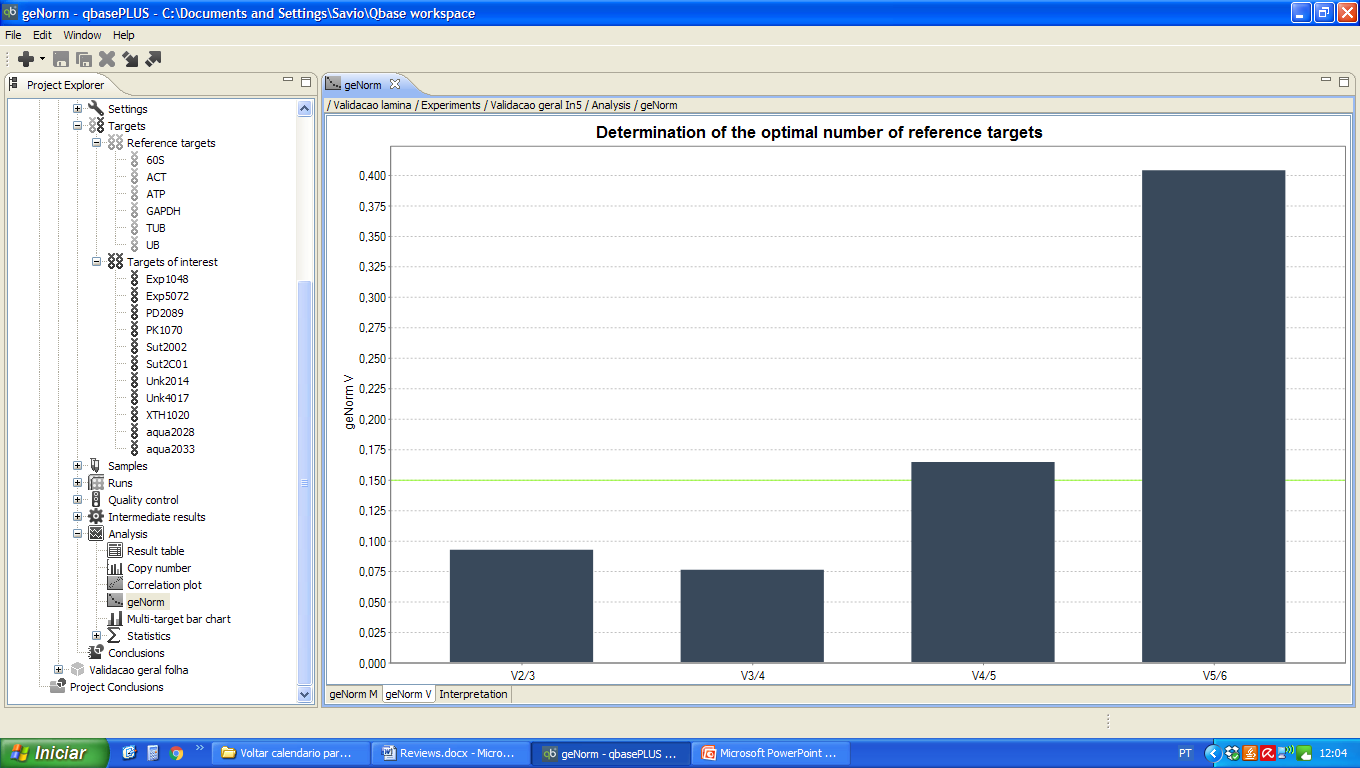

Supplement: Supplementary file 4 — Supplementary material 4 (DOCX 5002 kb) [file 11103_2016_434_MOESM4_ESM.docx]
