# Supplementary material for: Co-expression network analysis reveals transcription factors associated to cell wall biosynthesis in sugarcane
Source: Plant Mol Biol. 2016 Jan 28;91:15–35. doi: 10.1007/s11103-016-0434-2 (PMC4837222; doi:10.1007/s11103-016-0434-2)

**Online Resource 5. qPCR analysis of six genes in the lignin biosynthesis pathway in the intermediate internodes of a different set of plants from those used in the microarray assays (A).** Different letters at the top of each bar indicate different means by one-way ANOVA followed by Tukey’s test. Error bars=SEM; N=3; p<0.05. PAL, Phenylalanine ammonia lyase; 4CL, 4-coumatare-CoA ligase; COMT, caffeic acid 3-O methyltransferase; F5H, ferulate 5-hydroxylase; C4H, cinnamate 4-hydroxylase;CCR, cinnamoyl-CoA reductase. B, GeNorm results are shown as M-value (average expression stability) graph, V-value (pairwise variation) graph and thresholds are shown as a green line in each graph. GeNorm interpretation for each tissue analyzed is shown as well. Eight endogenous controls were tested to identify the best ones for data normalization.

**A**


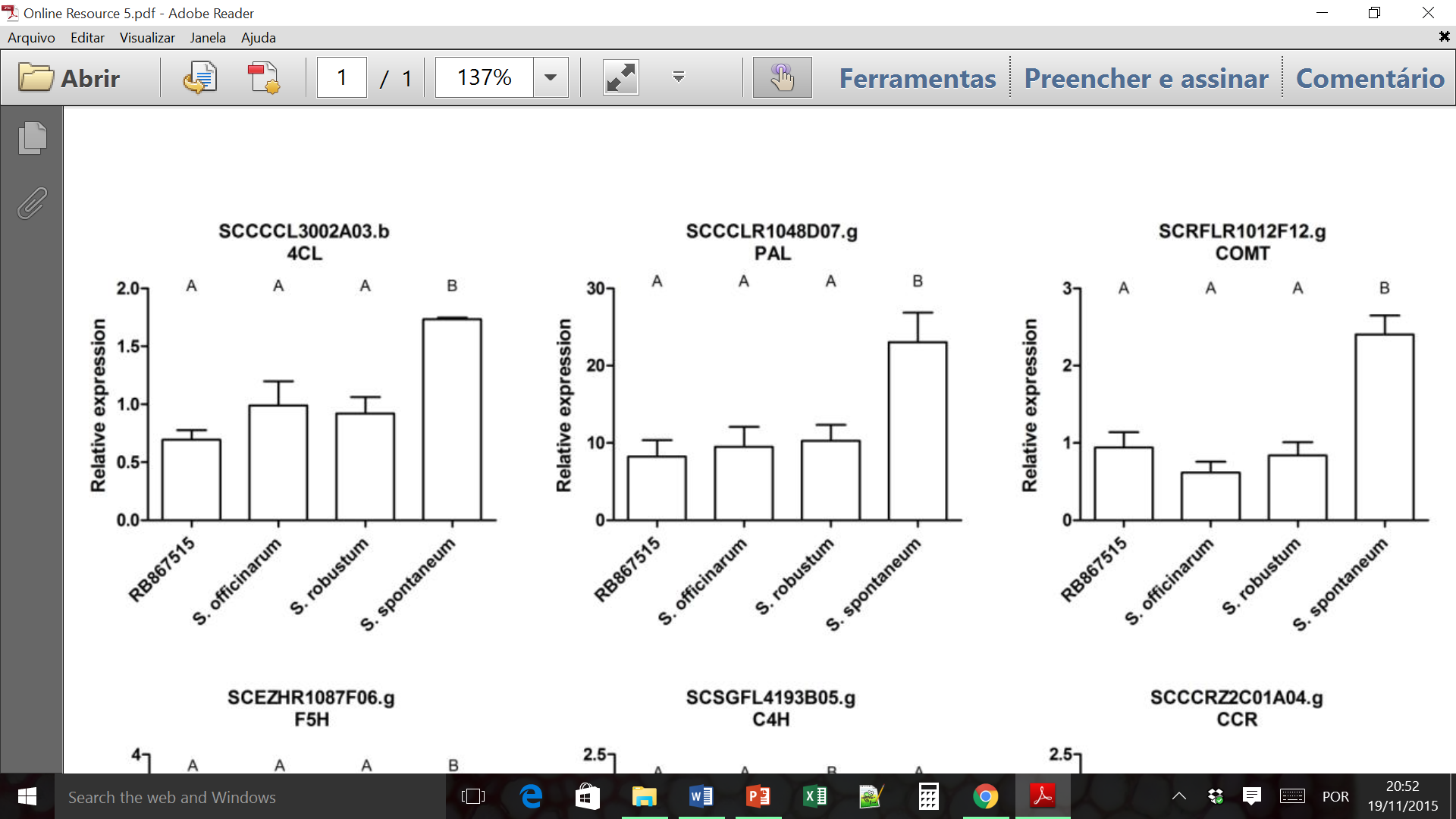

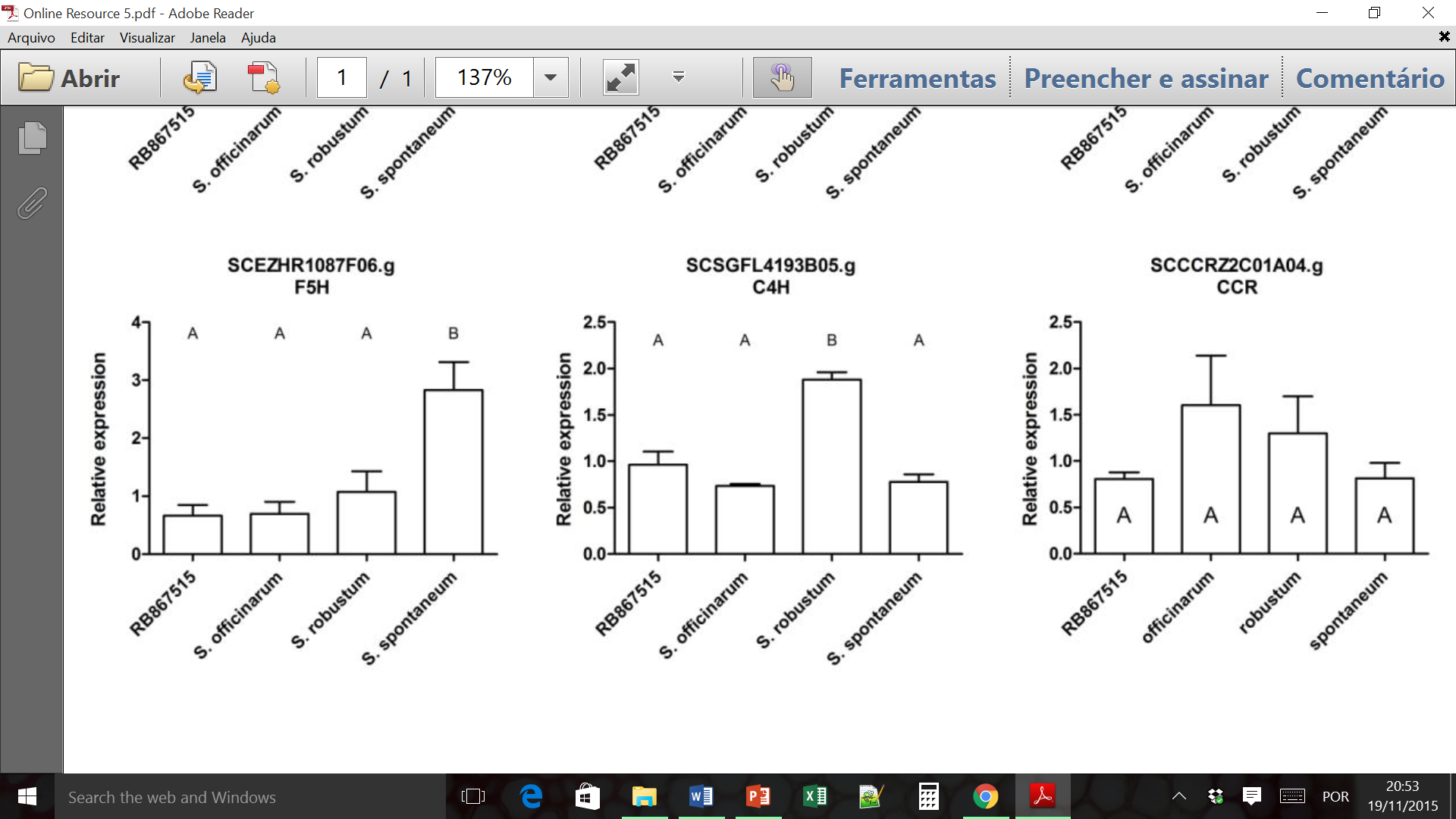


**B**


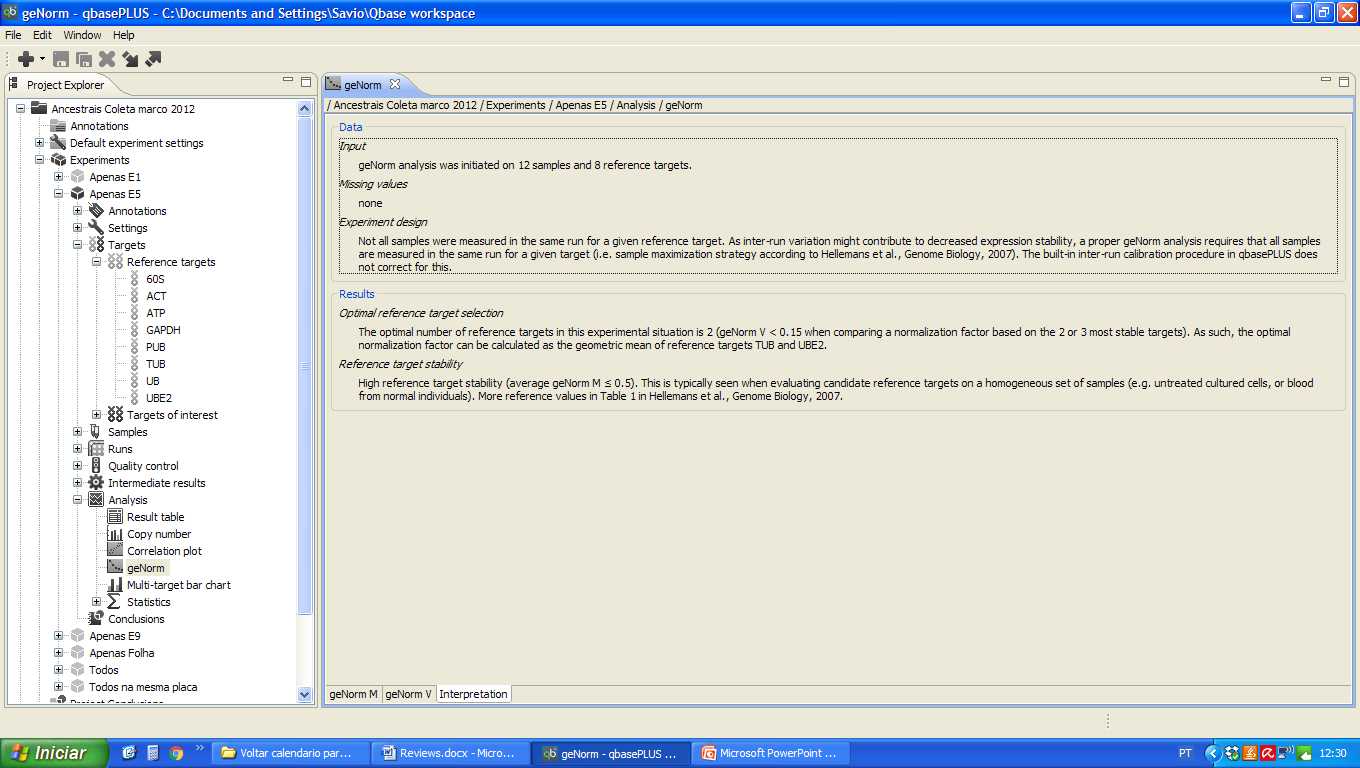

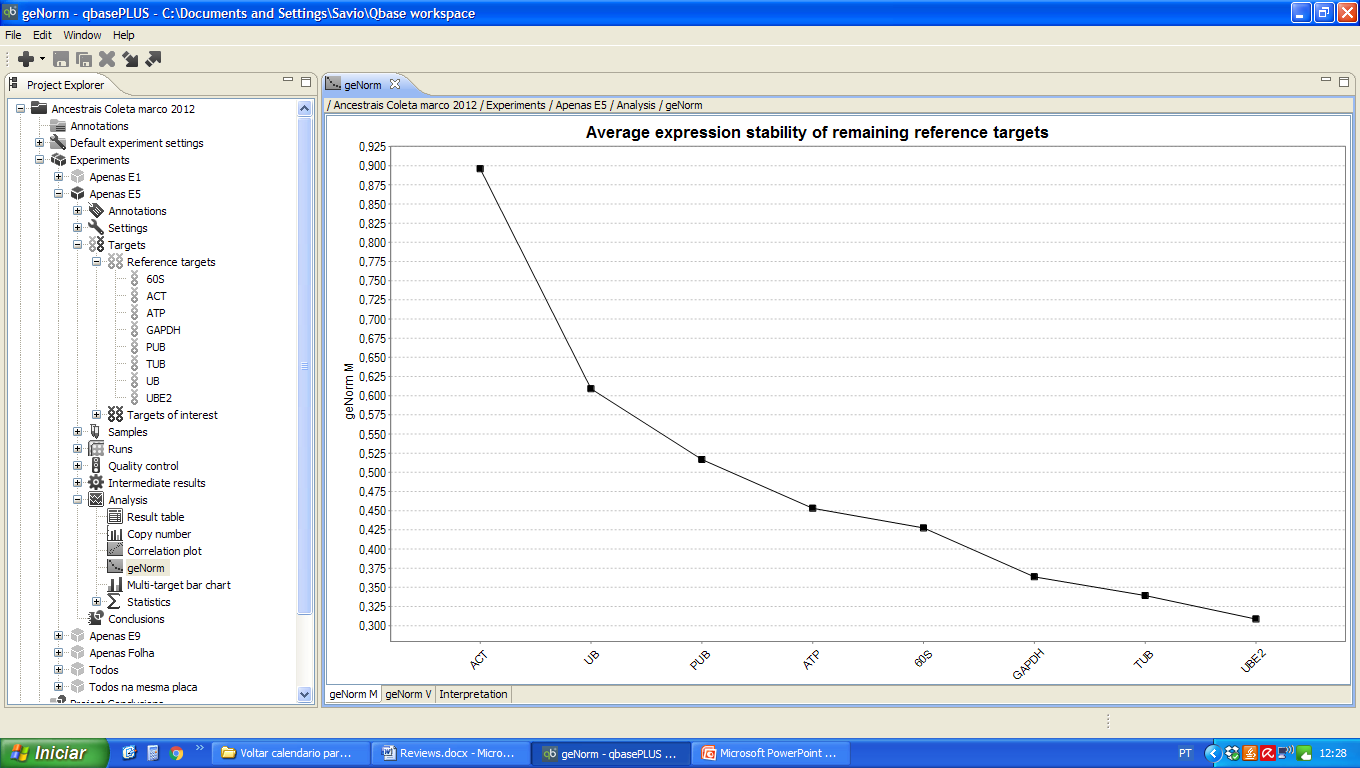

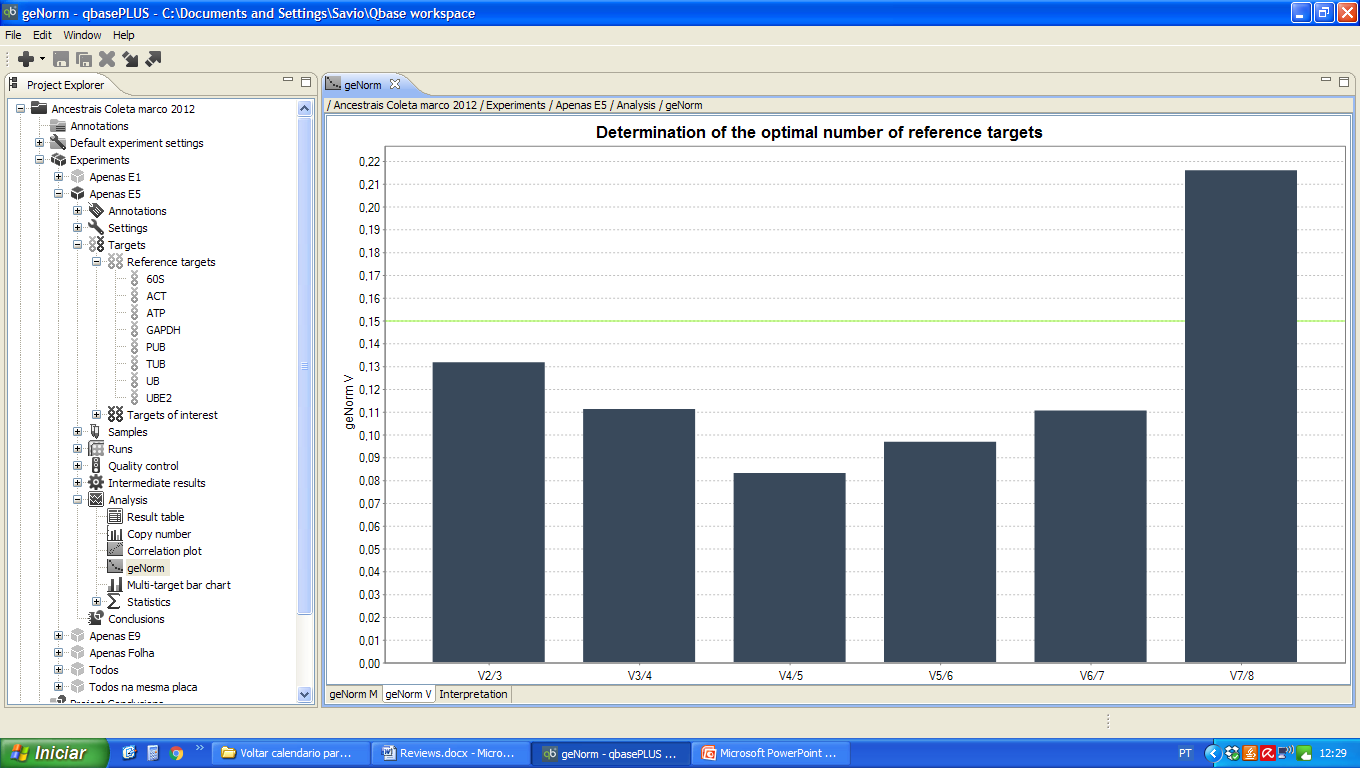

Supplement: Supplementary file 5 — Supplementary material 5 (DOCX 3547 kb) [file 11103_2016_434_MOESM5_ESM.docx]
