# Supplementary material for: Co-expression network analysis reveals transcription factors associated to cell wall biosynthesis in sugarcane
Source: Plant Mol Biol. 2016 Jan 28;91:15–35. doi: 10.1007/s11103-016-0434-2 (PMC4837222; doi:10.1007/s11103-016-0434-2)
Supplement: Supplementary file 6 — Supplementary material 6 (DOCX 279 kb) [file 11103_2016_434_MOESM6_ESM.docx]

Online Resource 8. Phylogenetic analysis of rice (Os) and sugarcane (Sc) MYB transcription factors (TFs). A, Maximum Parsimony tree of rice and sugarcane MYBs. B, inset of the subtree (black thick bar in A) containing the *OsMYB14*, which are closely-related to the Arabidopsis secondary cell wall activating *AtMYB46* [[1](#_ENREF_1)]. MYBs present in rice [[1](#_ENREF_1)] and sugarcane (Figure 7, this work) cell wall co-expression network are highlighted by red and green dots, respectively. Protein sequences were obtained from Grassius database (<http://grassius.org/>) and aligned using ClustalW within MEGA 5 [[2](#_ENREF_2)] using default parameters. Tree was created by maximum parsimony method (1000 bootstraps) using the close neighbor interchange on random trees MP search method





**

**

1. Hirano K, Aya K, Morinaka Y, Nagamatsu S, Sato Y, et al. (2013) Survey of Genes Involved in Rice Secondary Cell Wall Formation Through a Co-Expression Network. Plant and Cell Physiology 54: 1803-1821.

2. Tamura K, Peterson D, Peterson N, Stecher G, Nei M, et al. (2011) MEGA5: molecular evolutionary genetics analysis using maximum likelihood, evolutionary distance, and maximum parsimony methods. Mol Biol Evol 28: 2731-2739.
